# The human cerebral cortex is neither one nor many: neuronal distribution reveals two quantitatively different zones in the gray matter, three in the white matter, and explains local variations in cortical folding

**DOI:** 10.3389/fnana.2013.00028

**Published:** 2013-09-02

**Authors:** Pedro F. M. Ribeiro, Lissa Ventura-Antunes, Mariana Gabi, Bruno Mota, Lea T. Grinberg, José M. Farfel, Renata E. L. Ferretti-Rebustini, Renata E. P. Leite, Wilson J. Filho, Suzana Herculano-Houzel

**Affiliations:** ^1^Instituto de Ciências Biomédicas, Universidade Federal do Rio de Janeiro, Cidade UniversitáriaRio de Janeiro, Brasil; ^2^Instituto Nacional de Neurociência Translacional, MCT/CNPqSão Paulo, Brasil; ^3^Grupo de Estudos em Envelhecimento Cerebral da Faculdade de Medicina da Universidade de São PauloSão Paulo, Brasil; ^4^Instituto Israelita de Ensino e Pesquisa Albert EinsteinSão Paulo, Brasil; ^5^Departamento de Enfermagem Médico-Cirúrgica da Escola de Enfermagem da Universidade de São PauloSão Paulo, Brasil

**Keywords:** human, prefrontal cortex, occipital cortex, evolution, cortical expansion

## Abstract

The human prefrontal cortex has been considered different in several aspects and relatively enlarged compared to the rest of the cortical areas. Here we determine whether the white and gray matter of the prefrontal portion of the human cerebral cortex have similar or different cellular compositions relative to the rest of the cortical regions by applying the Isotropic Fractionator to analyze the distribution of neurons along the entire anteroposterior axis of the cortex, and its relationship with the degree of gyrification, number of neurons under the cortical surface, and other parameters. The prefrontal region shares with the remainder of the cerebral cortex (except for occipital cortex) the same relationship between cortical volume and number of neurons. In contrast, both occipital and prefrontal areas vary from other cortical areas in their connectivity through the white matter, with a systematic reduction of cortical connectivity through the white matter and an increase of the mean axon caliber along the anteroposterior axis. These two parameters explain local differences in the distribution of neurons underneath the cortical surface. We also show that local variations in cortical folding are neither a function of local numbers of neurons nor of cortical thickness, but correlate with properties of the white matter, and are best explained by the folding of the white matter surface. Our results suggest that the human cerebral cortex is divided in two zones (occipital and non-occipital) that differ in how neurons are distributed across their gray matter volume and in three zones (prefrontal, occipital, and non-occipital) that differ in how neurons are connected through the white matter. Thus, the human prefrontal cortex has the largest fraction of neuronal connectivity through the white matter and the smallest average axonal caliber in the white matter within the cortex, although its neuronal composition fits the pattern found for other, non-occipital areas.

## Introduction

The notion that the human being is extraordinary, an exception to evolutionary rules that apply to other primates, has pervaded studies of the neuroanatomical bases of human cognition. Ever since the initial description that the prefrontal cortex represents a larger percentage of the cortex in humans (29%) than chimpanzees (17%) and the rhesus monkey (11.5%; Brodmann, 1912), the higher cognitive skills attributed to primates have often been explained by the larger relative expansion of the neocortex in this order (Jerison, 1985), and in particular of the frontalmost, associative portion of the cortex, which would have attained its apex in humans (Bush and Allman, 2004; Smaers et al., 2010). Though recent studies using MRI showed that the human frontal cortex as a whole, and its gray matter in particular, do not deviate from the allometric rule that applies to other large primates (Semendeferi et al., 2002; Schoenemann et al., 2005; Smaers et al., 2011), the human prefrontal cortex does appear to deviate from that of other species in having more underlying white matter than expected for its gray matter volume (Schoenemann et al., 2005).

While seemingly supportive of a particular enlargement of the prefrontal cortical white (but not gray) matter, these studies do not address how cortical volume relates to numbers of neurons in the gray matter, or of fibers in the white matter. More problematically, their usual interpretation assumes an even distribution of neurons across the cortical volume (Rockel et al., 1980), and a resulting direct relationship between cortical volume and numbers of neurons, such that cortical volume can be a valid proxy for numbers of neurons across cortical regions. The same applies to the white matter: a larger-than-expected white matter volume only indicates a singular morphology of human prefrontal cortex if white matter fiber composition and relationship to neurons in the overlying gray matter are homogeneous throughout the cortex. However, the distribution of neurons in the human cerebral cortex and its relationship to white matter volume have never been examined systematically. To the contrary, a recent examination of the distribution of neurons across the cortical surface of non-human primates found that neuronal densities vary widely across locations (Collins et al., 2010). Similarly, a recent study of human and non-human white matter suggested that average axonal caliber is not constant across areas, increasing from frontal to occipital areas (Caminiti et al., 2009).

Our recent findings that the human cerebral cortical gray matter contains the number of neurons expected for a primate cortex of its mass (Azevedo et al., 2009), and the expected corresponding white matter volume (Herculano-Houzel et al., 2010), indicate that this structure is not extraordinary in its overall cellular composition (Herculano-Houzel, 2012a). However, these findings for the cerebral cortex as a whole do not exclude the possibility of different allometric rules applying to different cortical regions within the human cortex. One, two or many different allometric rules would be indicative of different developmental scenarios leading to the formation of the adult cerebral cortex as follows. An extreme scenario would be a single relationship between local cortical gray volume and number of neurons throughout the cerebral cortex, indicative of a single mechanism by which neurons are added to different regions and differentiate with similar average neuronal size, regardless of areal identity (which would then be determined by other factors, such as patterning of inputs and outputs), consistent with the protocortex theory (O'Leary, 1989; Dehay and Kennedy, 2007). The other extreme scenario would be purely local, area-specific relationships between cortical volume and number of neurons, such that no overall relationship would emerge across functional or topological areas. That would be consistent with a developmental mechanism of locally-regulated addition of neurons to different regions of the cortex, consistent with the protomap model (Rakic, 1988; Dehay and Kennedy, 2007). A similar principle would apply to the white matter, whose composition, average fiber caliber and relationship to numbers of gray matter neurons could follow a single, shared relationship throughout the cortex, be locally-specific, with no discernible relationship across areas, or show intermediate patterns.

Determining how neurons and white matter are distributed in the human cerebral cortex is important not only to address the issues of prefrontal cortex uniqueness and developmental mechanisms that lead to cortical formation, but also to shed light on the evolutionary mechanisms of cortical expansion and to inform necessary updates of computational models of cortical organization. Evo-devo and computational models of cortical organization have long assumed a homogeneous composition of the cerebral cortex gray and white matter (for instance, Rakic, 1988; Prothero, 1997; Zhang and Sejnowski, 2000; Karbowski, 2003) as well as other characteristics, such as a single relationship between neuronal density and cortical volume across species (Haug, 1987), and a constant number of neurons per cortical surface area (Rockel et al., 1980). However, we and others have shown that the distribution of neurons per cortical surface area is not homogeneous neither across species (Haug, 1987; Stolzenburg et al., 1989; Poth et al., 2005; Herculano-Houzel et al., 2008) nor within individual cortices (Collins et al., 2010; Cahalane et al., 2012). We have also shown that there is no single relationship between neuronal density and cortical mass across species, a diversity that makes the human cortex remarkable, but not an outlier, in its large number of neurons in a relatively small cortical volume [Herculano-Houzel et al., 2006, 2007, 2011; Herculano-Houzel, 2011a; Azevedo et al., 2009; Sarko et al., 2009; reviewed in Herculano-Houzel (2012a)]. The analysis of the distribution of neurons and other, mostly glial cells along the human cerebral cortex allows us to address how these parameters are related to others that are known to vary across the cortical surface: folding index (Zilles et al., 1988), cortical thickness, and the number of neurons beneath a unit of surface area (Collins et al., 2010).

## Materials and methods

To analyse the distribution of neurons and other (mostly glial) cells along the human cerebral cortical gray and white matter, we chose not to attempt to separate functional areas due to the lack of macroscopic criteria that would allow us to delimitate all areas in a manner that would be precise, reproducible, and enable future comparisons to other primate species. Instead, we opted for a systematic analysis of the distribution of cells along serial coronal sections of the entire cerebral cortex in the anteroposterior axis. Besides allowing a direct comparison to other primate species (MG and SHH, in progress), this approach allows the comparison of the prefrontal cortex (defined as all cortex that is anterior to the corpus callosum along the AP axis; Schoenemann et al., 2005) and of the occipital pole with the intermediate parietal, frontal and temporal regions. Additionally, due to the large variability in the absolute size and position of cortical areas across human individuals (Andrews et al., 1997), we chose to analyze a single human cortex, which is compatible with our goal of examining how several parameters related to cortical morphology relate to the distribution of neurons across cortical areas within a single cortex. Thus, while restricting our study to a single individual does not address the possibility of the confounding variable of individual differences in the distribution of neurons along the cerebral cortex, which is a fascinating theme that deserves further investigation in and of itself, it does allow the study of the several issues related to the distribution of neurons within a single cortex that we address here.

### Subject

We analyzed the distribution of cells in the right cerebral cortex of a 65-year-old human female who died of non-neurological causes and had no known risk factors for brain diseases such as diabetes, hypertension, drinking, or smoking. The brain was donated to this study by a next of kin, and was collected via the Brain Bank of the Brazilian Aging Brain Study Group (Grinberg et al., 2007), which is supplied by the autopsy service of the city of São Paulo (Serviço de Verificação de Óbitos da Capital), University of São Paulo, Brazil. Information provided by the family in a structured interview raised no concerns about cognitive or other behavioral abnormalities. Anatomical examination of the brain ruled out microvascular disease and other brain abnormalities that might affect the distribution of neurons in the cortex. This project was approved by the Ethics Committees of both that University and the Federal University of Rio de Janeiro, where the remainder of the study was executed. The brain was fixed by perfusion of the carotid and basilar arteries with 4% buffered paraformaldehyde and post-fixed by immersion in the same solution for 48 h.

### Morphometry

**Figure 1:**
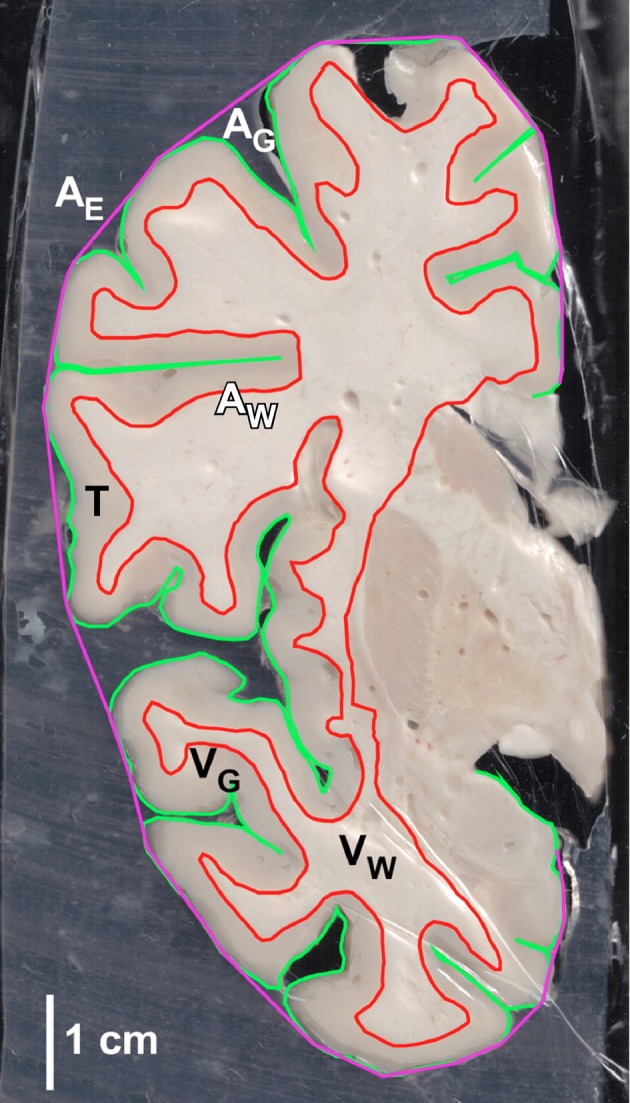
**Example of coronal section of the human cerebral cortex with reconstructions of outlines for determination of total cortical area (A_G_), exposed cortical area (A_E_), white-gray matter interface area (A_w_), gray matter volume (V_G_) and white matter volume (V_W_), and gray matter thickness (T)**.

After removal of the meninges and superficial vascularization, the cerebral cortical hemisphere was embedded in agar 3% and cut into a series of 101 coronal sections of 2 mm each in the anteroposterior axis with a large-capacity industrial deli cutter (Filizola, São Paulo, Brazil). The plane of section was perpendicular to the longest axis of the cortical hemisphere. The thickness of each section was verified with a pachimeter and found to vary by less than 0.01 mm. Sections were then digitalized in a scanner (1200 dpi) and analyzed in Canvas X software (ACDSystems) to determine, for each section, the total pial surface perimeter (P_G_) and the area of gray matter in the coronal plane (S_G_); the perimeter of the white-gray matter interface (P_W_, which does not include the interface with the striatum) and the area of subcortical white matter external to the striatum in the coronal plane (S_W_; Figure 1).

Determining cortical surface area and volumes by multiplying coronal perimeters and areas by section thickness (2 mm) would underestimate areas and volumes at both extremities of the cerebral cortex, due to its curved shape. Instead, we used formulas that calculate area and volume from a series of sections in a manner that is sensitive to the inclination of the cerebral cortical surface. These formulas are based on the measurements of the gray or white matter perimeters in the coronal plane, P, and of the coronal area occupied by the gray or white matter in the section, S. The formulas that calculate total gray and white matter surface areas A_G_ and A_W_ and volumes V_G_ and V_W_ for each section in a series of coronal areas and perimeters S_0_, S_1_, S_2_ … and P_0_, P_1_, P_2_ … obtained as above are the following (see supplementary information):

$$\begin{array}{l} A_{n}={\left\{{\left(S_{n}-S_{n-1}\right)}^{2}+{\left[h\left(P_{n}+P_{n-1}\right)\text{​}/2\right]}^{2}\right\}}^{1/2}\\ V_{n}=h\left[S_{n}+S_{n-1}+{\left(S_{n}\cdot S_{n-1}\right)}^{1/2}\right]/3 \end{array}$$

where *h* = 2 mm (section thickness). Total values of A_G_, A_W_, V_G_, and V_W_ are obtained by summing all local measurements.

We calculated the local cortical thickness (T) of the gray matter as the ratio V_G_/A_G_. The local gyrification index (GI), a measure of the degree of cortical gyrification of a coronal section (Zilles et al., 1988), was calculated by first tracing the exposed surface of the gray matter (the smallest surface of gray matter that did not enter sulci) to determine A_E_ in a section, then determining the ratio A_G_/A_E_ for that section. This method, however, provides biased estimates of GI at the extremities of the cortex (frontal, temporal, and occipital poles), where sections become increasingly tangential to the surface, yielding GI values that are biased toward unity. For this reason, those sections at the extremities with GI values below 2.0, which is the lower limit of the range of GI values found for the remainder of the AP axis, were discarded in the analysis of GI covariation (see below).

After scanning, each section had its cortical white and gray matter separated with a scalpel, using the small amount of resistance that the white matter offers to being pulled from the gray matter exactly at the gray/white matter interface. We processed separately the white and gray matter of the temporal lobe (regions ventral to the lateral sulcus, and excluding the amygdala), and the gray matter of the insula (defined as the cortical portion occupying the fundus of the lateral sulcus, parallel to the outer cortical surface). The remaining white and gray matter of each section (including the capsula extrema, medial to the insula) were processed collectively as “dorsal cortex” (that is, non-temporal regions). The hippocampus was removed from all sections. In some sections, portions of V1, easily identified by the presence of the Stria de Gennari, were processed separately. All values for V1, where it was identified, were included in the corresponding sections for analysis.

We recorded the mass (g) of the gray and white matter of each analyzed structure to determine cell densities in a manner that is comparable with our previous studies. To determine the total number of neuronal and other (mostly glial) cells in each structure, we then applied the Isotropic Fractionator (Herculano-Houzel and Lent, 2005), which transforms the anisotropic cerebral tissue into an isotropic suspension of cell nuclei whose density in the suspension of known volume can promptly be determined.

### Numbers of neurons and other cells

After homogenizing each structure of interest and collecting it as a suspension containing all cell nuclei, nuclei were stained with DAPI (4′,6-diamidino-2-phenylindole, diluted 20–50x from a stock solution of 20 mg/L, *Invitrogen*, USA) and the suspension adjusted to a round, exact volume. The density of nuclei in the suspension, and therefore the total number of nuclei in the suspension and in the original structure, were determined using a Neubauer-improved chamber under a fluorescent microscope (Zeiss, Germany). Typically, four samples of each suspension were counted, which yields an average number of nuclei for that structure with a coefficient of variation below 10%.

Once total number of cells was established in a structure, the neuronal fraction was determined by immunocytochemistry to the neuronal marker NeuN (Mullen et al., 1992), and the number of other (mostly glial) cells was obtained by subtracting the number of neurons from the total number of cells in the structure. Briefly, a 1 ml sample of the isotropic suspension was centrifugated, washed twice in phosphate-buffered saline (PBS), and re-suspended in boric acid 0.2M, pH 9.0 for 45 min at 70°C for heat-induced epitope retrieval. The sample was then washed twice in PBS and incubated in PBS containing anti-mouse NeuN (*Invitrogen*, Eugene, OR) in a dilution of 1:200 for 2 h at room temperature. After another wash, the sample was ressuspended in PBS containing 10 mg/L DAPI, 10% goat serum and anti-mouse Alexa-Fluor 546 secondary antibody (*Invitrogen*, Eugene, OR) diluted 1:200 and incubated at room temperature for another 2 h. After a final wash in PBS, we counted at least 500 nuclei per sample to obtain the percentage of neurons in each structure by all DAPI stained nuclei that also expressed NeuN.

Each gray matter structure analyzed had percentages of NeuN-positive nuclei counted independently in two or three samples by three researchers (PFMR, LVA and MG). The first count was done in identified samples, ordered by section, by PFMR, and the recounts where done blindly and in random order by the two other researchers. We found a good correlation between the initial count and the recounts (Spearman rank correlation, ρ = 0.6011, *p* < 0.0001) and the total number of neurons in each structure was then calculated based on the mean of the percentages obtained. Isotropic suspensions of free cell nuclei from all structures were preserved in an anti-freeze solution (Herculano-Houzel, 2012b) and stored at −20°C for future studies.

### Mathematical analysis of the alometric cellular scaling rules

All results are reported across sections, with each datapoint representing values for an individual section. All relationships between parameters were analyzed across sections for the entire cerebral cortex in order to identify patterns common to cortical regions. The main morphometric parameters are illustrated in Figure 1. We analyzed pairwise relationships between gray matter volume (V_G_, in mm^3^), gray matter surface area (A_G_, in mm^2^), gyrification index for the gray matter (GI), numbers of neurons in the gray matter (N_G_), numbers of other cells in the gray matter (O_G_), densities of neurons and other cells in the gray matter (D_N_ and D_O_), the ratio between other cells and neurons in the gray matter (O/N), gray matter thickness (T, in mm), numbers of neurons per unit area of gray matter surface (N/A, in N/mm^2^), white matter volume (V_W_, in mm^3^), surface area of the gray-white matter inferface (A_W_, in mm^2^), and numbers of other cells in the white matter (O_W_). Neurons eventually found in the white matter were disconsidered, as at the moment it cannot be ascertained whether they are contaminants from the gray matter or interstitial neurons in the white matter. However, these were a small percentage of all neurons in each portion of gray matter analyzed, and are unlikely to affect the results in any significant manner. As mentioned above, correlation analyses of GI with other parameters excluded those sections at the prefrontal, temporal, and occipital poles with GI < 2.0 (sections 1–6, 26–28, and 97–101, respectively).

All statistical analyses were performed with JMP 9.0 (SAS, USA). Principal component analysis was performed with the entirety of the dataset, across all variables and cortical sections, to determine what parameters contribute most to variations along the AP axis. Correlations were calculated using Spearman coefficients, which do not require assumptions about normality in the dataset. Our initial null hypothesis, in all pairwise correlations, is that there is no relationship between the parameters across cortical sections. When a correlation was observed, we then tested whether the variation across the dataset was best explained by a single correlation (or power function) that applies to the entire AP axis, or by two or more functions applied to the different cortical zones identified anatomically (prefrontal, dorsal and temporal cortex) or visually (occipital) as explained below. Power functions and linear relationships in pairwise comparisons are characterized by their exponents or slopes, reported here with the corresponding standard error, 95% confidence interval and *p*-value. As the power functions that applied to different non-occipital regions (for gray matter analyses) or to different non-occipital, non-prefrontal regions (for white matter analyses) were found to be similar to one another, we do not report them separately here; rather, in those cases we report only the joint power functions that apply across regions.

All correlation and power analyses were thus performed for the entire dataset as well as separately for sets of cortical regions and combinations thereof: prefrontal, dorsal, temporal, insular, parietal, and occipital. Prefrontal cortex was identified as the cortex found in all sections anterior to the corpus callosum (Schoenemann et al., 2005), that is, sections 1–17. Insular and temporal regions were defined anatomically, as described above, and were found in sections 32–51 and 26–57, respectively. “Dorsal” cortex refers to sections posterior to the prefrontal cortex as well as the portion of cortex that lies above the lateral sulcus in all sections where a temporal cortex was also identified, and extends from sections 18 to 57. “Parietal” cortex (sections 58–72) refers to all sections posterior to the end of the lateral sulcus and anterior to “occipital” cortex, and thus also includes ventral cortical areas that are known not to be strictly parietal. Only the “occipital” region was not identified based on purely anatomical, *a priori* criteria, but rather on the analysis of the relationship between numbers of cells and tissue volume. As shown in Figures 6–8, we found that all datapoints from section 73 until section 101, and only these, were outliers in the relationship between gray matter volume and number of neurons, constituting what appeared like a different cortical “zone” with a distinct relationship between these variables that was however common to sections 73–101. These sections were thus marked as “occipital” for these and all latter analyses. All Figures and analyses thus employed the same separation of datapoints across cortical regions, always identified by the same criteria as defined here, and color-coded in the same way: prefrontal in red, temporal in black, dorsal in orange, insular in gray, parietal in blue, and occipital in light green. V1 datapoints are shown in dark green, but all values obtained for these portions of V1 were included in the “occipital” datapoints for analysis.

## Results

The cerebral cortex hemisphere analyzed had a total gray matter volume of 276,361 mm^3^, a total white matter volume of 222,251 mm^3^, average cortical thicknesses between 2 and 4 mm per section, total gray matter pial surface area of 94,050 mm^2^, total white-gray matter interface surface area of 78,722 mm^2^, and an exposed pial surface of 42,619 mm^2^, which yields an average folding index of 2.21. These values are closely comparable with oft-cited volumes and surface areas in the literature (Stephan et al., 1981; Hofman, 1985), and well within the range of a recent MRI study with 486 individuals that found the surface area of both cortical hemispheres to range between 135,000 and 190,000 mm^3^, gray matter volume to range between 350,000 and 550,000 mm^3^, and overall cortical thickness to vary between 2.4 and 2.8 mm (Winkler et al., 2010). This cortical hemisphere had a total of 4.63 billion neurons and 24.33 billion other cells, 15.49 billion of these located in the white matter. While these total values are well within the range of the other hemispheres studied previously in the lab (6.18 ± 1.72 billion neurons in the gray matter and 19.88 ± 2.83 billion other cells in the white matter), they are certainly underestimations of the total cellular composition of this hemisphere due to inevitable tissue loss in the slicing procedure, which leaves visible tissue residue on the blade, and should not be used for analysis. However, as tissue loss is presumably equal across all sections, the analysis of the distribution of cells across the cortical surface and volume and its relationship to morphological parameters is not compromised.

To examine the relationship between local morphometric characteristics and numbers of cells, all analyses were performed across coronal sections in the antero-posterior (AP) axis. Each datapoint in the graphs presented thus corresponds to one coronal section or portion thereof, as detailed in the Methods. As mentioned above, total numbers of neurons in each region of the AP axis cannot be determined reliably here due to the inevitable loss of tissue in the sectioning. However, the relative number of cortical neurons found in each region can be calculated from the distribution of neurons. We find that the prefrontal cortex holds 7.6% of all cortical neurons; “dorsal” cortex, 27.1%; temporal cortex, 11.8%; insula, 1.3%; “parietal” cortex, 19.1%; and “occipital” cortex, 33.3% of all cortical neurons. Except for the occipital cortex, these percentages are fairly close to the relative volume of gray matter found in each region: 10.0% of the entire cortical gray matter in prefrontal cortex; 32.9% in “dorsal” cortex; 14.0% in temporal cortex; 1.4% in the insula; 18.7% in “parietal” cortex; and 22.9% in “occipital” cortex.

### Principal component analysis

The joint multivariate analysis across all cortical sections of all parameters in the dataset (section number, V_G_, A_G_, N_G_, O_G_, V_W_, A_W_, O_W_, A_E_, D_N_, D_O_, O/N, N/A, T, GI, n, a, n.N.a/A_E_) results in four principal components that explain 92% of the variation in the dataset. The first component accounts for 42% of the variation (*p* < 0.0001, eigenvalue 7.6131) and is loaded by N_G_, O_G_, O_W_, V_G_, V_W_, A_G_, A_W_, A_E_—that is, by the total numbers of cells and the volume and surface area that they compose in the sections. The second component, which accounts for another 27% of the variation (*p* < 0.0001, eigenvalue 4.8356), is further loaded by section number, D_N_, D_G_, O/N, n, and N/A—that is, the relationships between volumes, surface areas, and numbers of cells, as well as the connectivity fraction and part of the brain. The third component accounts for another 10% of the variation (*p* < 0.0001, eigenvalue 1.7544) is loaded by GI and n.N.a/AE, that is, cortical folding. Finally, the fourth component, which accounts for an added 13% of the variation (total, 92% of the variation; *p* = 0.0003, eigenvalue 1.4019) is further loaded by T and a, that is, cortical thickness and the average caliber of the fibers in the white matter. These findings suggest that cortical thickness and folding are results of the distribution of neurons along the cortical volume and surface, and that position along the AP axis is an important factor in determining at least some of the relationships amongst the parameters. We next report pairwise correlations across these parameters, guided by our previous analyses of the cellular composition of the cerebral cortex and by our connectivity-driven model of cortical folding (Mota and Herculano-Houzel, 2012).

### Variation along the AP axis

**Figure 2:**
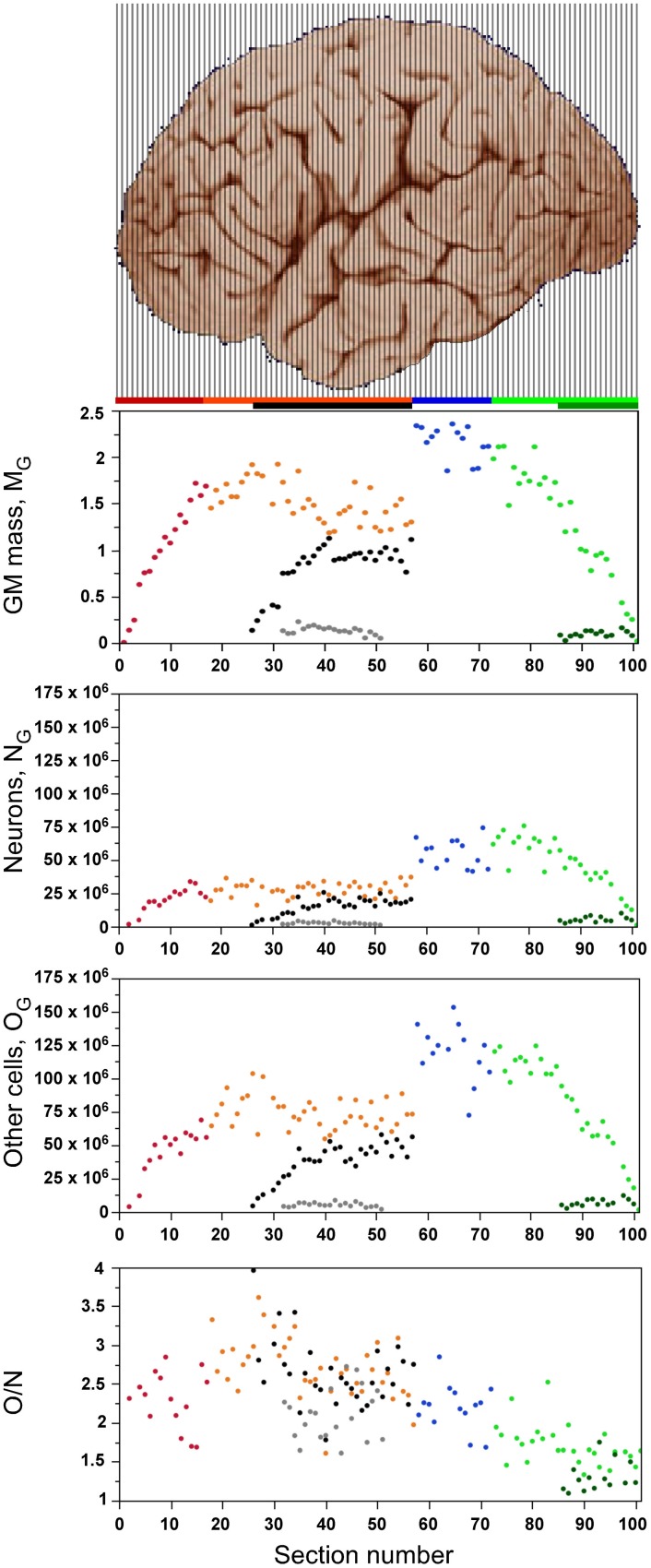
**Distribution of cells and tissue. Graphs show the distribution of gray matter mass (M_G_, top panel), number of neurons (N_G_, second panel), number of non-neurons (other cells, O_G_, third panel) and O/N ratio (bottom panel) along the anteroposterior axis of the cerebral cortex (anterior to the left). Each point represents one individual coronal section**. Cortical regions are aligned with the image of cerebral cortex illustrating the 101 coronal sections, and data points are color-coded as shown in the bar: prefrontal (red), dorsal (orange), “parietal” (blue), “occipital” (light green), temporal (black), insula (gray) and V1 (dark green). V1 values are contained in the corresponding “occipital” data points. Image of the human cerebral cortex from the University of Wisconsin and Michigan State Comparative Mammalian Brain Collections (www.brainmuseum.org).

As expected from the variation in gray matter mass along the AP axis (Figure 2, top) due to the curved shape of the cerebral cortex, local numbers of neurons and other cells vary greatly across sections (Figure 2). In all sections, the gray matter consists of fewer neurons (N_G_) than other cells (O_G_, shown in the same scale in Figure 2), such that the O/N ratio is larger than 1.0 in all sections (Figure 2, bottom), but smaller in the posteriormost sections than in the others, as discussed below.

**Figure 3:**
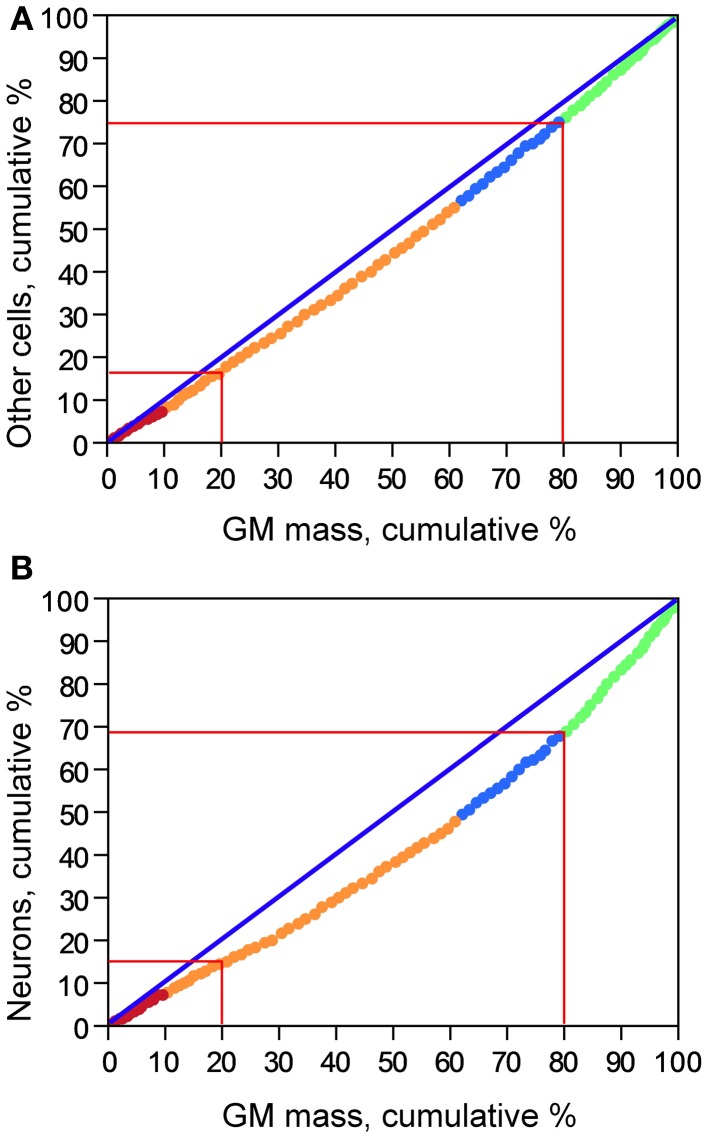
**Cumulative distribution of other cells (A) and of neurons (B) along the cortical gray matter**. X axis: percentage of cumulative gray matter mass along the anteroposterior axis; Y axis: percentage of cumulative number of other cells **(A)** or of neurons **(B)** along the anteroposterior axis. Data points represent individual coronal sections and are color-coded as indicated in Figure 2.

The cumulative distribution of other cells in the gray matter along the AP axis is relatively close to the expected for a homogeneous distribution, as shown by its close (although not perfect) correspondence to the cumulative distribution of gray matter mass (Figure 3A), such that the 20% anteriormost portion of the cortical gray matter holds about 15% of all other cells in the gray matter, and the posteriormost 20% portion of the gray matter holds about 25% of all other cells in the gray matter. In comparison, the cumulative distribution of neurons deviates further from the cumulative distribution of gray matter mass along the AP axis (Figure 3B); while the 20% anteriormost portion of the gray matter contains about 15% of total number of cortical neurons, the 20% posteriormost cortical mass concentrates over 30% of all cortical neurons. This difference suggests that the distribution of neurons in the human cerebral cortex is skewed toward the more posterior, occipital regions, while the distribution of other cells in the gray matter is less so.

### Distribution of neuronal and other cell densities

**Figure 4:**
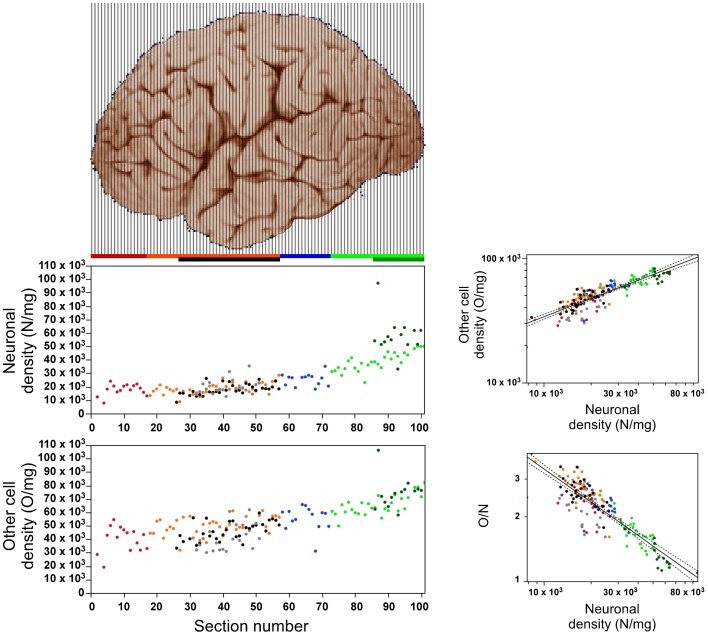
**Relationship between local variations in neuronal density and in other cell density**. Left, neuronal (top) and other cell densities (bottom) increase along the anteroposterior axis along the posterior half of the cerebral cortex (anterior is to the left). Right, top: Density of other cell density varies across all sections as a power function of neuronal density with exponent 0.485 ± 0.024 (*p* < 0.0001). Exponents within each region: prefrontal, 0.784 ± 0.151 (*p* < 0.0002); dorsal, 0.467 ± 0.068 (*p* < 0.0001); “parietal,” 0.762 ± 0.202 (*p* = 0.0027); “occipital,” 0.378 ± 0.092 (*p* = 0.0003); temporal, 0.498 ± 0.078 (*p* < 0.0001); insula, 0.700 ± 0.126 (*p* < 0.0001); V1, 0.514 ± 0.107 (*p* = 0.0004). Right, bottom: The O/N ratio varies across all sections as a power function of neuronal density with expoent −0.515 ± 0.024 (*p* < 0.0001). Data points are color-coded as before: prefrontal (red), dorsal (orange), “parietal” (blue), “occipital” (light green), temporal (black), insula (gray) and V1 (dark green). Power laws and 95% confidence intervals are plotted for the entire dataset.

Both neuronal and other cell densities vary along the AP axis (Figure 4). Overall, neuronal density varies 5x (between approximately 10,000 and 50,000 N/mg), and other cell density varies 3x (approximately 30,000–90,000 O/mg). This variation, however, is not gradual along the entire axis; rather, both densities remain stable for the first 40 sections (neuronal density, ρ = 0.019, *p* = 0.9102; other cell density, ρ = 0.314, *p* = 0.0587), increasing from this point forward until they reach peak values in the occipital pole (neuronal density, ρ = 0.883, *p* < 0.0001; other cell density, ρ = 0.741, *p* < 0.0001, Figure 4). Neuronal densities are similar across insular, temporal, and the corresponding “dorsal” cortex (ANOVA Tukey *p* > 0.05; Table 1), and vary in concert along the AP axis across the temporal and “dorsal” portions of the same sections (ρ = 0.654, *p* < 0.0001), increasing along the AP axis (temporal cortex, ρ = 0.654, *p* < 0.0001). In contrast, the “parietal” cortex exhibits significantly higher neuronal densities than the regions anterior to it (ANOVA Tukey *p* < 0.05; Table 1). Neuronal densities are highest in the occipital cortex than in all other more anterior areas (Table 1), and even higher in anatomically identified area V1 than in the surrounding non-V1 occipital cortex, reaching an average 58,162 ± 13,640 N/mg across all portions of V1 analyzed.

**Table 1:** **Mean neuronal density, other cell density and other cell/neuron ratio across cortical regions**.

**Cortical region**	**Neuronal density ± *SE***	**Other cell density ± *SE***	**O/N ratio ± *SE***
Prefrontal	17,742 ± 4,240	40,055 ± 9,543	2.291 ± 0.366
Frontal	18,768 ± 3,996	49,660 ± 6,587	2.713 ± 0.391
Temporal	18,013 ± 4,124	46,245 ± 7,092	2.638 ± 0.430
Insula	19,897 ± 5,628	40,999 ± 9,774	2.102 ± 0.333
Parietal	25,394 ± 4,595^***^	55,565 ± 9,017	2.208 ± 0.295
Posterior	38,960 ± 6,901^*^	65,347 ± 7,359^*^	1.712 ± 0.263^**^
V1	58,162 ± 13,641^*^	74,214 ± 11,325^*^	1.306 ± 0.191^**^

*Cortical regions (first column), mean neuronal density ± standard error (second column), mean other cell density ± standard deviation (third column), other cell/neuron ratio ± standard deviation (fourth column)*.

* significantly different from all other cortical regions, ANOVA p < 0.05.

** , significantly smaller than all other cortical regions, ANOVA p < 0.05.

*** , significantly larger than all other cortical regions except the posterior, V1 and insula, ANOVA p < 0.05.

Across the entire extension of the cerebral cortex, variations in other cell density are correlated with variations in neuronal density, such that other cell density increases as a power function of neuronal density with an exponent of 0.485 ± 0.024 (*p* < 0.0001; Figure 4). Thus, despite the 3–5× variation and the cell densities peaking in the occipital cortex and V1, all cortical regions, including area V1, share the same relationship between neuronal and other cell densities.

The O/N ratio, which is a good approximation of the glia/neuron ratio, varies by approximately 4× along the AP axis (Figure 4). As predicted by our model of cortical evolution (Herculano-Houzel, 2011b), the O/N ratio varies across all portions of the human cerebral cortex as a single power function of local neuronal density with an exponent of −0.515 ± 0.024 (95% CI −0.563–0.467, *p* < 0.0001; Figure 4).

### Cortical thickness, surface area and gyrification index

**Figure 5:**
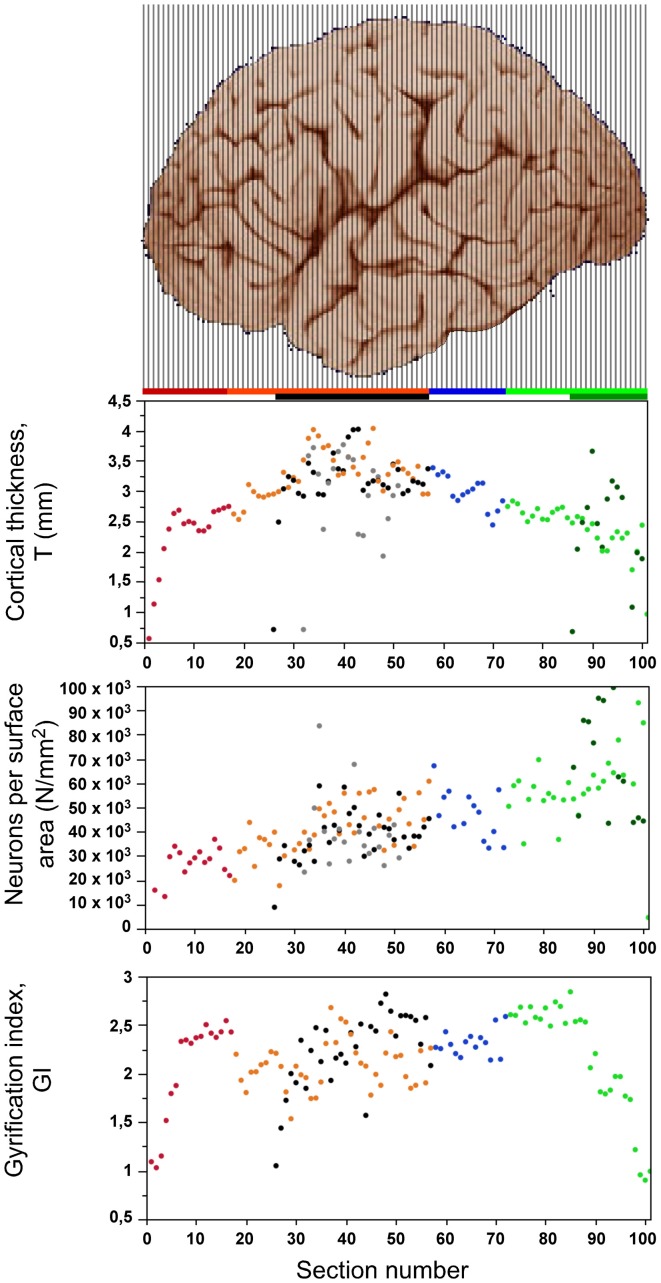
**Distribution of cortical thickness (top), number of neurons under 1 mm^2^ of cortical surface (center) and gyrification index (bottom) along the anteroposterior axis**. Data points are aligned with the respective cortical regions illustrated in the cerebal cortex above (image from the University of Wisconsin and Michigan State Comparative Mammalian Brain Collections, www.brainmuseum.org). Data points are color-coded as shown in the bar: prefrontal (red), dorsal (orange), “parietal” (blue), “occipital” (light green), temporal (black), insula (gray) and V1 (dark green). V1 values are contained in the corresponding “occipital” data points.

The distribution of average cortical thickness of the coronal sections has an inverted U-shaped along the AP axis, with thinnest gray matter in both extremities, and thickest in the central region of the dorsal, temporal and insular cortices (Figure 5, top). Most of the cortical gray matter along the AP axis has a thickness between 2 and 4 mm, with smaller average thicknesses in the anteriormost sections (Figure 5). Some sections of V1 have higher cortical thickness values than the remaining, non-V1 occipital region.

The number of neurons per mm^2^ of gray matter surface area (N/A) varies at least 3x along the AP axis in a homogeneous manner (ρ = 0.751, *p* < 0.0001 throughout the cortex), and not mainly in the posteriormost region like neuronal density (Figure 5, middle). The lowest values of around 20,000 neurons/mm^2^ are found in the frontal pole, and the highest values are found in the occipital pole (up to 70,000 neurons/mm^2^), and in V1 in particular (over 90,000 neurons/mm^2^). Even if the entire posterior cortex is excluded (green datapoints in Figure 5), the N/A ratio is still found to increase significantly across the AP axis (ρ = 0.509, *p* < 0.0001).

After excluding the prefrontal, temporal, and occipital poles from the analysis, the local gyrification index (GI) varies between 1.53 and 2.87 across cortical sections (Figure 5, bottom), with an inverted U-shaped distribution that resembles the distribution of cortical thickness along the AP axis (Figure 5, top). However, as described below, there are several inconsistencies between the two distributions, such as a discontinuity between prefrontal (red) and dorsal (orange) cortex along the axis, and a steep decrease in GI in the occipital areas that does not mirror the smoothly decreasing cortical thickness in the sections.

### Gray matter volume relationships

In theory, if a different relationship between gray matter volume and number of neurons applied to each small region of the cerebral cortex, a plot of local gray matter volume by number of neurons in that cortical section should have the appearance of a disorganized cloud of data. On the other hand, if a single mechanism governed the construction of the gray matter volume, then all datapoints in the said plot should cluster along a single line.

**Figure 6:**
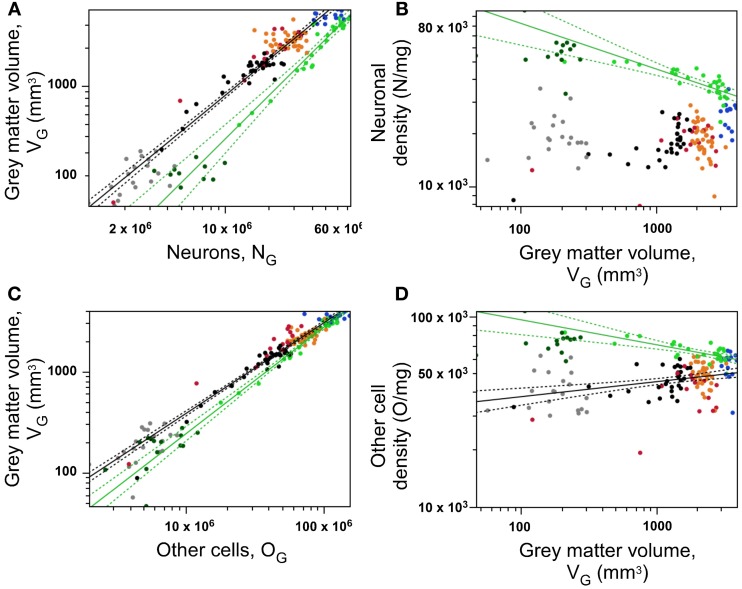
**Neurons are distributed differently across occipital and non-occipital cortical zones, but similarly within each zone. (A)** Gray matter volume within each section varies as a shared function of the number of neurons in the section within occipital cortex (light green points) and across non-occipital areas (all other points). **(B)** Neuronal density decreases significantly with increasing gray matter volume across coronal sections within occipital cortex (green), but not across non-occipital areas. (**C**), Gray matter volume within each section varies as a shared function of the number of other cells in the section within occipital cortex (light green points) and across non-occipital areas (all other points). **(D)** density of other cells decreases significanty with increasing gray matter volume across sections within occipital cortex (green), and increases across sections of non-occipital cortex (other colors). Data points are color-coded as before: prefrontal (red), dorsal (orange), “parietal” (blue), “occipital” (light green), temporal (black), insula (gray) and V1 (dark green). V1 values are contained in the corresponding “occipital” data points and V1 data points are not included in the fits. The fitted power functions, where significant, are shown separately for each cortical zone (occipital in green, and non-occipital in black), with 95% confidence intervals shown as dashed lines.

We find an intermediate result of two clearly distinct relationships between local gray matter volume (V_G_) and number of neurons (N_G_) in the cerebral cortex: one that applies to the “occipital” cortex (green), and another that applies to all other regions (all other colors; Figure 6A). In the occipital region, V_G_ increases supralinearly with N_G_ with an exponent of 1.234 ± 0.080 (95% CI 0.959–1.288, *p* < 0.0001), while in the remaining cortical regions, including prefrontal cortex, V_G_ increases sublinearly with N_G_ with an exponent of 0.929 ± 0.022 (95% CI 0.884–0.972, *p* < 0.0001). In addition to the different exponents, the two distributions are offset so that for a given cortical volume, more neurons are found in the occipital cortical areas compared to any other cortical region.

The different relationships for occipital and other cortices between gray matter volume and number of neurons imply that neuronal density (D_N_) varies systematically with cortical volume (that is, across sections) but in different manners across the occipital cortex and across all other cortical regions. Indeed, D_N_ in the occipital cortex decreases significantly with increasing gray matter volume with an exponent of −0.205 ± 0.036 (*p* < 0.0001), which indicates that the V_G_ × N_G_ relationship is indeed supralinear, and not linear. Thus, average neuronal size appears to be smaller in the occipital regions than in the others, and to increase with cortical volume within the occipital region (that is, it decreases along the AP axis within occipital cortex). In contrast, D_N_ increases significantly with V_G_ across the non-occipital cortical regions with an exponent of 0.211 ± 0.038 (*p* < 0.0001; Figure 6B), although, as seen above, this increase probably does not translate into a gradual change in average neuronal size along the AP axis of the non-occipital cortical areas, given that neuronal densities only begin to increase in the parietal cortex (Figure 2, Table 1).

As observed for numbers of neurons, we find two mutually excludent relationships between local gray matter volume and number of other cells in occipital and non-occipital cortical regions (Figure 6C). Within the occipital region, cortical gray matter volume varies approximately linearly with its number of other cells, with an exponent of 1.058 ± 0.039 (95% CI 0.979–1.138, *p* < 0.0001), while in the remaining cortical regions as a whole, including temporal and insular cortex, it varies with an exponent of 0.901 ± 0.017 (95% CI 0.866–0.935, *p* < 0.0001; Figure 6C). In line with our observation that variations in local densities of neuronal and other cells are correlated, we find the same pattern of variation in other cell density with gray matter volume: D_O_ decreases with increasing gray matter volume in the occipital cortex, with an exponent of −0.130 ± 0.028 (*p* < 0.0001), and increases in non-occipital cortex with an exponent of 0.079 ± 0.019 (*p* < 0.0001; Figure 6D).

### Gray matter surface relationships

**Figure 7:**
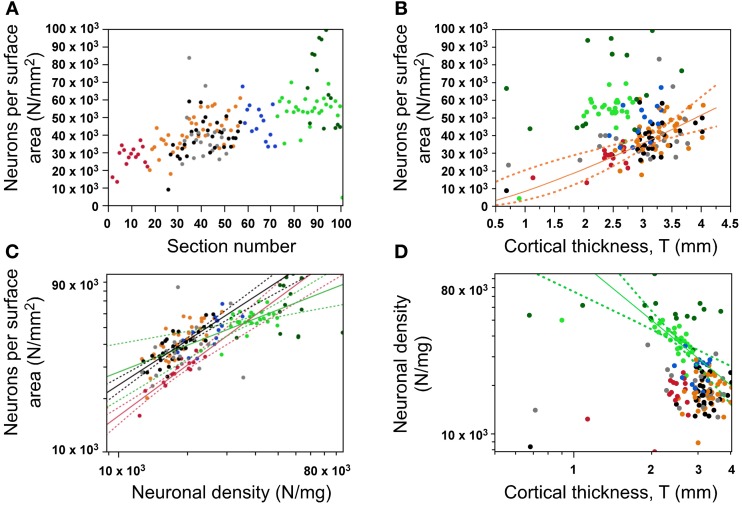
**Variation in number of neurons per surface area (N/A, or N/mm^2^). (A)** Variation of the number of neurons under a cortical surface (N/A) along the anteroposterior axis; **(B)** Variation of N/A as a function of cortical thickness (only statistically significant in dorsal areas); **(C)** Variation in N/A as different functions of neuronal density across cortical zones (occipital in green, prefrontal in red, all others in black); **(D)** Variation in neuronal density is only significantly correlated with cortical thickness in the occipital cortex. Data points are color-coded as before: prefrontal (red), dorsal (orange), “parietal” (blue), “occipital” (light green), temporal (black), insula (gray) and V1 (dark green). V1 values are contained in the corresponding “occipital” data points and V1 data points are not included in the fits. The fitted power functions, where significant, are shown separately for each cortical zone (occipital in green, and non-occipital in black), with 95% confidence intervals shown as dashed lines.

Since A_G_ = V_G_/T and V_G_ = N_G_/D_N_, the N/A ratio is expected to vary as a function of D_N_, T, or a combination of both these parameters. The finding that N/A, D_N_, and T vary with different patterns along the AP axis (Figures 4, 5, 7A) already indicates that there is no single relationship among these parameters that applies to the entire cortical surface. Indeed, cortical thickness is not correlated with N/A across all cortical regions (ρ = 0.071, *p* = 0.4007), although N/A does vary significantly with cortical thickness in the dorsal cortex (ρ = 0.508, *p* = 0.0010; all others, *p* > 0.05; Figure 7B, orange). This lack of a single relationship indicates that local variations in N/A are not explained by variations in cortical thickness. On the other hand, variations in neuronal density are correlated with local variations in N/A across regions in the cerebral cortex, although again not in a single fashion, but with three different relationships: N/A varies as a power function of neuronal density with an exponent of 0.498 ± 0.132 in the occipital region (*p* = 0.0009), with an exponent of 0.874 ± 0.067 in the prefrontal region (*p* < 0.0001), and with an intermediate exponent of 0.715 ± 0.079 in the remaining cortical regions as a whole (*p* < 0.0001; Figure 7C). With these three different relationships for different cortical regions, a same value of N/A corresponds to higher neuronal densities in the occipital than in non-occipital, non-prefrontal cortices; and similar neuronal densities correspond to smaller N/A ratios in prefrontal than in non-prefrontal, non-occipital cortices (Figure 7C, compare red and green points with all other).

In line with these observations, neuronal density varies coordinately with cortical thickness only within the occipital region, as a power function of exponent −1.316 ± 0.267 (*p* < 0.0001; Figure 7D). Although D_N_ and T might seem to be correlated across all datapoints (ρ = −0.391, *p* < 0.0001), this correlation is due to the occipital datapoints, and does not survive the exclusion of these (ρ = −0.052, *p* = 0.5807). The lack of a common relationship between cortical thickness and neuronal density across the cerebral cortex suggests that a locally thicker cerebral cortical wall does not necessarily result from smaller neuronal densities (that is, from a larger average neuronal size; Figure 7D), even though this relationship does seem to apply within the occipital cortex.

**Figure 8:**
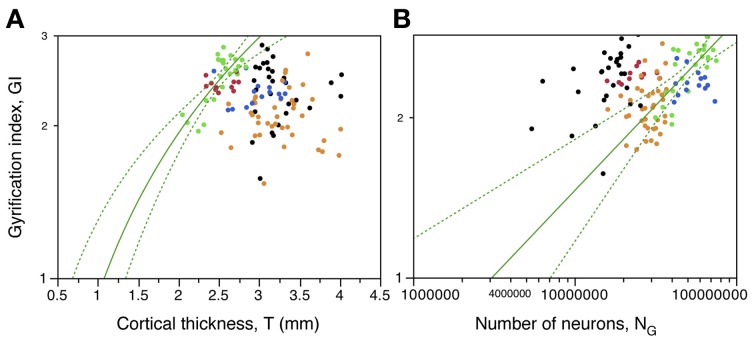
**Local variations in cortical folding are not universally correlated with cortical thickness or with local numbers of neurons. (A)** Local gyrification index is only significantly correlated with cortical thickness in the occipital cortex (green). **(B)** Local gyrification index varies as different power functions of the number of neurons in the prefrontal, temporal and occipital regions. Data points are color-coded as before: prefrontal (red), dorsal (orange), “parietal” (blue), “occipital” (light green), temporal (black), insula (gray) and V1 (dark green). V1 values are contained in the corresponding “occipital” data points and V1 data points are not included in the fits. The fitted power functions, where significant, are shown separately for each cortical zone (prefrontal in red, occipital in green, and non-occipital in black), with 95% confidence intervals shown as dashed lines.

We next test the hypotheses that the degree of cortical folding (1) is influenced by cortical thickness, which might offer different degrees of mechanical resistance to folding, and (2) is a simple function of numbers of cortical neurons. Excluding from analysis those sections corresponding to the prefrontal, temporal, and occipital poles, we find a poor correlation between gyrification index (GI) and cortical thickness across all sections along the AP axis (ρ = −0.239, *p* = 0.0087; Figure 8A). However, only within the occipital regions is there a significant correlation (ρ = 0.590, *p* = 0.0024), and in the opposite direction from the expected if a thicker cortex opposed folding, that is, we find a positive correlation between local cortical thickness and GI within the occipital cortex (Figure 8A, green). For all other regions, correlations do not reach significance (prefrontal, “dorsal,” temporal and “parietal,” *p* = 0.6893, 0.8262, 0.3560, and 0.6025, respectively). These findings indicate strongly that local variations in cortical thickness do not determine the degree of cortical gyrification along the surface of the cerebral cortex, although these parameters are related in the occipital cortex.

Although there is a weak significant correlation between GI and number of neurons across all sections of the cerebral cortex (ρ = 0.211, *p* = 0.0230, excluding prefrontal, temporal, and occipital poles; Figure 8B), there is not a simple relationship between GI and number of neurons across the cerebral cortex as a whole. There is no significant correlation between GI and N_G_ in the “dorsal” (ρ = 0.216, *p* = 0.1875) or “parietal” (ρ = −0.024, *p* = 0.9346) regions. However, GI varies concertedly with N_G_ in the occipital region (ρ = 0.618, *p* = 0.0013), as a power function of exponent 0.320 ± 0.064 (95% CI 0.192–0.448, *p* < 0.0001); in the temporal region (ρ = 0.404, *p* = 0.0328), as a power function of exponent 0.152 ± 0.066 (95% CI 0.020–0.284, *p* = 0.0304); and in the prefrontal region (ρ = 0.618, *p* = 0.0013), although the fit to a power function here does not reach significance (*p* = 0.2090; Figure 8B). The distribution of GI against local numbers of neurons also shows that for a similar number of neurons, the gray matter is more folded in temporal than in prefrontal cortex, and more in prefrontal than in occipital cortex (Figure 8B).

The lack of simple correlations between local GI along the AP axis and numbers of neurons or cortical thickness suggests that variations in cortical folding within an individual cortex are not a simple function of local numbers of neurons or cortical thickness. We have recently proposed a model that predicts gray matter folding as a consequence of characteristics of not only gray, but also, and predominantly, white matter, such as connectivity and average axonal caliber (Mota and Herculano-Houzel, 2012). We next examine how these properties vary in the subcortical white matter along the AP axis in relationship to the distribution of neurons in the gray matter, then test whether our model predicts the local variations in cortical folding along the human cerebral cortex.

### White matter volume relationships

**Figure 9:**
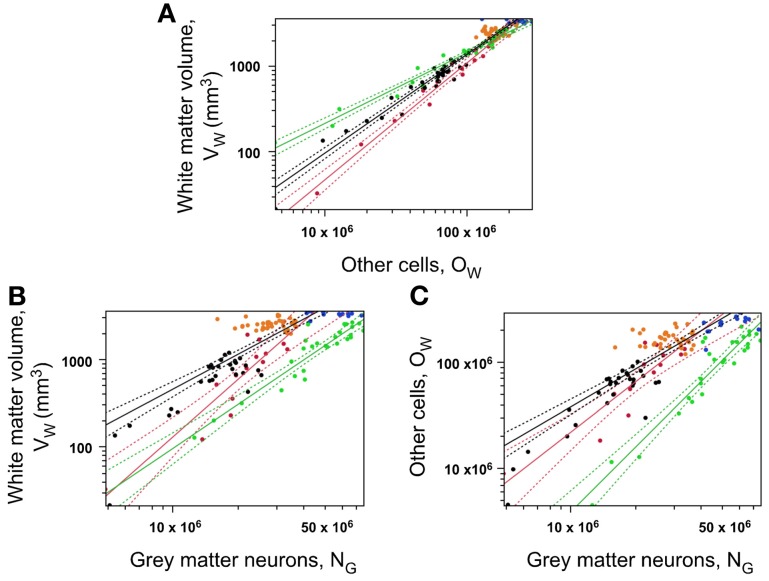
**Local white matter volume varies as different functions of numbers of neurons across prefrontal, occipital, and non-occipital cortical zones, but similarly within each zone. (A)** Local white matter subcortical volume is a different function of its number of other cells in the occipital (green), prefrontal (red) and remaining cortical regions (black curve). **(B)** Local white matter subcortical volume is a different function of the number of neurons in the gray matter in the same section across the occipital (green), prefrontal (red) and remaining cortical regions (black curve). Notice that the same white matter volume is associated with larger numbers of neurons in the occipital than in any other cortical zone. **(C)** Number of other cells in the white matter (O_W_) is a different function of the number of neurons in the gray matter in the same section across the occipital (green), prefrontal (red) and remaining cortical regions (black curve). Data points are color-coded as before: prefrontal (red), dorsal (orange), “parietal” (blue), “occipital” (light green), temporal (black), insula (gray) and V1 (dark green). V1 values are contained in the corresponding “occipital” data points and V1 data points are not included in the fits. The fitted power functions are shown separately for each cortical zone (prefrontal in red, occipital in green, and non-occipital in black), with 95% confidence intervals shown as dashed lines.

While a single relationship relates neuronal to other cells throughout the cortex and two relationships relate gray matter volume to numbers of neurons (one for occipital and one for non-occipital cortex), we find that, as observed for N/A, there are three different patterns relating white matter volume (or number of other, mostly glial, cells) to numbers of gray matter neurons: one for occipital, one for prefrontal, and one for non-occipital, non-prefrontal cortex (Figure 9). White matter volume (V_W_) increases with the number of other cells in the white matter (O_W_) raised to non-overlapping exponents of 0.822 ± 0.031 in the “occipital” cortex (95% CI 0.760–0.884, *p* < 0.0001), 1.362 ± 0.063 in the prefrontal cortex (95% CI 1.236–1.488, *p* < 0.0001) and 1.148 ± 0.030 in the other cortical areas (95% CI 1.088–1.208; Figure 9A). Given that O_W_ is a good approximation for the number of oligodendrocytes in the white matter, and that this in turn is related linearly to total axon length L in the white matter (Barres and Raff, 1994), the non-overlapping distributions suggest that the fiber composition of the white matter differs across prefrontal, occipital, and intermediate cortical areas. Considering that L ~ O_W_, and knowing that V_W_ = L × *a* (where *a* is the average fiber caliber in the white matter), then V_W_ ~ O_W_ × *a*. The variation of O_W_ as a function of N_G_, O_W_ ~ N^ω^_G_, then allows us to calculate how average axon caliber *a* varies as a function of N^α^_G_, by rewriting the proportion V_W_ ~ O_W_ × *a* as a function of N^α^_G_ such that V_W_ ~ N^ν^_G_ ~ N^ω^_G_ × N^α^_G_.

The relationship V_W_ ~ N_G_, like V_W_ × O_W_, differs between prefrontal, occipital, and the intermediate areas, with non-overlapping exponents, ν, of 2.196 ± 0.287 (95% CI 1.622–2.770, *p* < 0.0001), 1.680 ± 0.127 (95% CI 1.426–1.934, *p* < 0.0001) and 1.471 ± 0.100 (95% CI 1.271–1.671, *p* < 0.0001; Figure 9B). This means that for similar numbers of neurons in the gray matter, the volume of the corresponding, underlying white matter is smaller in the occipital cortex than in prefrontal cortex, and smaller in the prefrontal cortex than in the non-occipital, non-prefrontal areas, at the same time that white matter volume increases much faster in prefrontal cortex than in occipital cortex as these gain neurons in the gray matter locally.

Likewise, the relationship O_W_ ~ N_G_ differs between prefrontal, occipital, and the intermediate areas, with non-overlapping exponents, ω, of respectively 1.576 ± 0.218 (95% CI 1.140–2.012, *p* < 0.0001), 2.026 ± 0.142 (95% CI 1.742–2.310, *p* < 0.0001) and 1.273 ± 0.082 (95% CI 1.109–1.437, *p* < 0.0001; Figure 9C). This means that for similar numbers of neurons in the gray matter, the number of other cells in the underlying white matter, which serves as a proxy for total length of myelinated axons, is much smaller in the occipital cortex than in prefrontal cortex, and smaller in the prefrontal cortex than in the non-occipital, non-prefrontal areas, at the same time that white matter volume increases much faster in occipital cortex than in prefrontal cortex as these gain neurons in the gray matter locally.

With these exponents ν and ω obtained empirically, the exponent α that describes how average axon caliber varies with numbers of neurons across the cerebral cortex can be determined as ν − ω, yielding 0.620 for the prefrontal cortex, 0.190 for the non-prefrontal, non-occipital areas, and −0.346 for the occipital areas. Since N_G_ first increases and then decreases along the AP axis (Figure 2), average axonal caliber in the white matter can be inferred to increase from the frontal to the occipital pole along the AP axis.

### White matter surface relationships

Just like the relationships between V_W_, O_W_ and N_G_ inform about the variation in average axonal caliber in the white matter along the AP axis, the relationship between A_W_ and N_G_ informs about the local fraction of gray matter neurons connected through the white matter, using our model according to which A_W_, the surface area of the white-gray matter interface, is proportional to the product n × N_G_ × *a*, where n is the connectivity fraction (the fraction of gray matter neurons connected through the white matter). A_W_ can be written as a function of N_G_ such that A_W_ ~ N^c^_G_ × N^1^_G_ × N^α^_G_ ~N^c + 1 + α^_G_. Since the exponent α is already known for each cortical region, the exponent c can be determined from the exponent of the empirical relationship A_W_ ~ N_G_.

**Figure 10:**
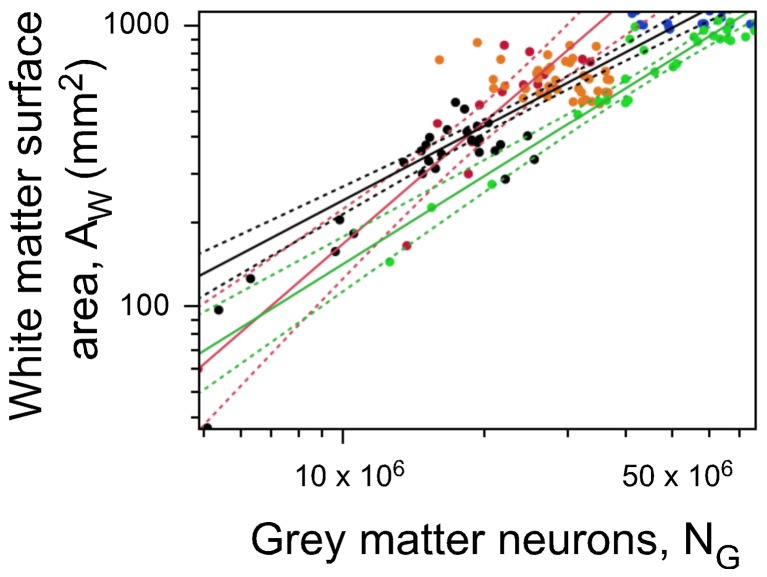
**White matter surface area is not a single function of numbers of neurons**. The gray-white matter surface area (AW) in each section is plotted against the number of gray matter neurons in that section. Prefrontal (red), occipital (green) and other regions (all other colors) exhibit different relationships between A_W_ and N, indicating that neurons are not distributed homogeneously over the white matter surface. Notice that the same white matter surface area is associated with larger numbers of neurons in the occipital than in any other cortical zone. Data points are color-coded as before: prefrontal (red), dorsal (orange), “parietal” (blue), “occipital” (light green), temporal (black), insula (gray) and V1 (dark green). V1 values are contained in the corresponding “occipital” data points and V1 data points are not included in the fits. The fitted power functions are shown separately for each cortical zone (prefrontal in red, occipital in green, and non-occipital in black), with 95% confidence intervals shown as dashed lines.

We find that A_W_ grows as different power functions of the number of cortical gray matter neurons N_G_ across cortical regions with non-overlapping exponents of 1.040 ± 0.069 in the “occipital” cortex (95% CI 0.898–1.183, *p* < 0.0001), 1.442 ± 0.158 in the prefrontal cortex (95% CI 1.098–1.786, *p* < 0.0001), and 0.877 ± 0.053 (95% CI 0.772–0.982, *p* < 0.0001) in the remaining non-occipital, non-prefrontal cortical regions (Figure 10). Besides the different exponents, we find that a same number of gray matter neurons is distributed over a smaller white matter surface in the occipital than in all other regions of the human cerebral cortex (Figure 10). This difference is expected from the higher neuronal densities found in the occipital cortex, but in our model, it is also expected from a smaller connectivity fraction in the occipital cortex.

Using the previously determined exponent of α and the empirically observed exponent relating A_W_ to N_G_, we show that the exponent c relating the connectivity fraction, n, to N_G_ is low in the prefrontal region (−0.178), even lower in the remaining non-posterior regions (−0.313), and high in the occipital region (0.386). Since N_G_ first increases and then decreases along the anteroposterior axis (Figure 2B), the fraction of cortical gray matter neurons sending or receiving projections through the white matter is inferred to decrease from frontal to occipital pole. Thus, the smaller A_W_ for a similar local N_G_ in the occipital cortex can be explained by a smaller connectivity fraction that compensates for a larger average axonal caliber in the occipital region compared to others.

**Figure 11:**
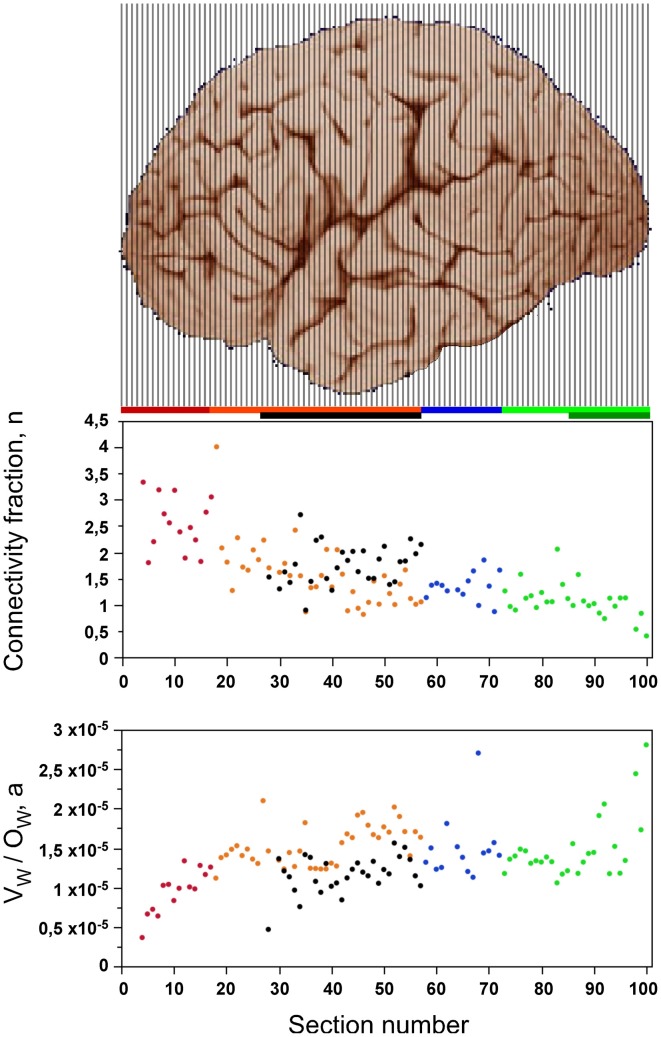
**Gray matter connectivity through the white matter decreases while average axonal caliber increases along the AP axis**. Top, estimate proportional to the connectivity fraction, n [derived from (A_W_/N_G_)^*^(O_W_/V_W_)], of all gray matter neurons that are connected through the white matter, either sending or receiving axons through it. Bottom, estimate proportional to the average axon caliber (derived from V_W_/O_W_) in the white matter. Each data point corresponds to one coronal section along the AP axis. Data points are color-coded as indicated by the bars: prefrontal (red), dorsal (orange), “parietal” (blue), “occipital” (light green), temporal (black), insula (gray) and V1 (dark green). Image from the University of Wisconsin and Michigan State Comparative Mammalian Brain Collections, www.brainmuseum.org.

How actual values of n and *a* change over the AP axis may be deducted mathematically using the previously described equations V_W_ ~ O_W_ × *a* and A_W_ ~ 2n × N_G_ × *a*, whose combination yields (I) *a* ~ V_W_/O_W_, and (II) n ~ A_W_/N_G_*a*. Substituting (I) into (II) it follows that n ~ (A_W_/N_G_)^*^(O_W_/V_W_). Since these are proportionalities, and not equalities, the quantities calculated in this way, shown in Figure 11, are not the absolute values of n and *a*, but are proportional to them. While originally formulated for the whole cortex (Mota and Herculano-Houzel, 2012), these equations still apply for individual sections within a cortex provided that, in the case of n, the total caliber of the outgoing fibers is approximately the same of the total caliber of the incoming fibers. We obtain a systematic reduction of cortical connectivity, n, across the anteroposterior axis of the cerebral cortex (Figure 11, top), and an increase in average axonal caliber, a, along the AP axis within the prefrontal cortex, and then again within the occipital cortex.

Notice that this simple model of the relationship between gray and white matter, n ~ (A_W_/N_G_)^*^(O_W_/V_W_), relates A_W_/N_G_ to the product n.*a*. Because A_W_ and A_G_ are strongly correlated (ρ = 0.7144, *p* < 0.0001), the number of neurons underneath a unit surface area of gray matter should thus vary across the cortical surface together with the product n.*a*. Indeed, we find that local variations in N/A are inversely correlated with the product n.a (ρ = −0.7144, *p* < 0.0001), confirming our model and its prediction that variations in the surface density of neurons along the cortical surface are due to the combined variation in local connectivity fraction and the average fiber caliber in the white matter.

### Local variations in cortical folding

Lastly, and given that local variations in cortical thickness and in numbers of neurons did not explain local variations in the degree of cortical folding, we sought to determine what parameters would best explain the latter. In particular, we wanted to examine our hypothesis that cortical folding varies as a consequence of the combination of numbers of neurons; the fraction of neurons that is connected through the white matter; tension along the axons; and average fiber caliber in the white matter, even though this hypothesis was originally formulated to account for variations in cortical folding across species (Mota and Herculano-Houzel, 2012). If our model is correct, then the folding of the gray matter surface is related to the folding of the white matter surface, F_W_, which can be written as N_G_ ~ N^(1 + c + α − 2^λ^)/3^, where λ is the exponent relating average fiber length in the white matter to numbers of neurons. Alternatively, given that variations in λ cannot be determined with confidence here because of the changing shape of the white matter along the AP axis, we can estimate F_W_ from the same model as the ratio N_G_.n.*a*/A_E_. Thus, if our model is correct and also applies to the local distribution of cortical folding within an individual, the ratio N_G_.n.a/A_E_, which describes the folding of the gray/white matter interface, should be the best predictor of the local variations in GI found within the human cortex.

**Figure 12:**
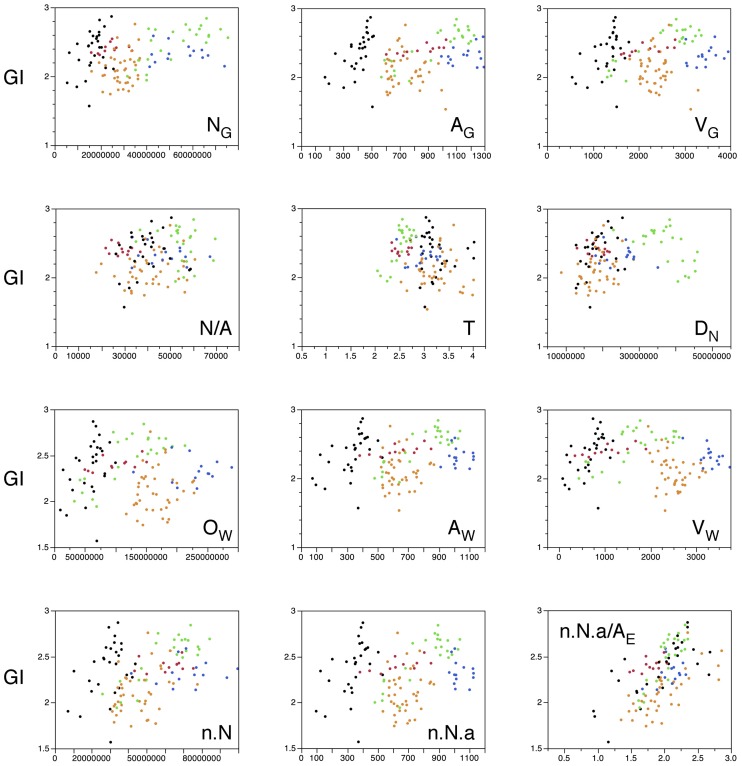
**Local variations in cortical folding are best explained by the folding index of the white matter**. Graphs show the local gyrification index, GI, plotted against different parameters: the number of neurons in the gray matter in that section (N_G_), the surface area of the gray matter in that section (A_G_), the volume of the gray matter in that section (V_G_), the number of neurons per mm^2^ of gray matter surface (N/A), average cortical thickness in the section (T), average neuronal density in the section (D_N_), number of other cells in the white matter of that section (O_W_), surface area of the gray-white matter interface in that section (A_W_), volume of white matter in that section (V_W_), the product n.N (which is proportional to the total number of neurons in the gray matter that are connected through the white matter), the product n.N.a (which is proportional to the surface area of the white matter), and the ratio n.N.a/AE (which is the value proportional to the folding of the white matter surface as estimated by our model; Mota and Herculano-Houzel, 2012). Each point represents one section. Data points are color-coded as before: prefrontal (red), dorsal (orange, including the insula), “parietal” (blue), “occipital” (light green), and temporal (black). Correlation coefficients are given in Table 2. Notice that the single parameter that best correlates with GI across all sections is the folding of the white matter surface as predicted by our model (Mota and Herculano-Houzel, 2012).

Indeed, we find that this is the case. Table 2 illustrates that, when only those sections without subcortical structures are considered (that is, the combination of prefrontal/temporal/occipital regions in our sample), the best predictor of the local variations in the degree of cortical folding is the degree of folding of the white matter, estimated by the ratio N_G_.n.a/A_E_ (ρ = 0.755, *p* < 0.0001), followed by the volume of white matter (ρ = 0.618, *p* < 0.0001) and by the number of other cells in the white matter (ρ = 0.599, *p* < 0.0001), even though the parameter A_G_ (gray matter surface) is, by definition, the one that is most closely related to GI (ρ = 0.513, *p* < 0.0001). In fact, the estimated degree of folding of the white matter is also the best predictor of local variations in GI even when all sections are analyzed, including those that accommodate subcortical structures (ρ = 0.615, *p* < 0.0001, Table 2; Figure 12, bottom right). Notice, in Figure 12, that the ratio N_G_.n.a/A_E_ is the only parameter significantly correlated with GI for which datapoints distributions for sections with (orange and blue datapoints) and without subcortical nuclei (red, black and green datapoints) are overlapping. We thus conclude that local variations in cortical folding along the entire surface of the human cerebral cortex are most closely related to properties of the white matter: its degree of folding, its volume, and the total length of myelinated axons therein (approximated by O_W_).

**Table 2:**
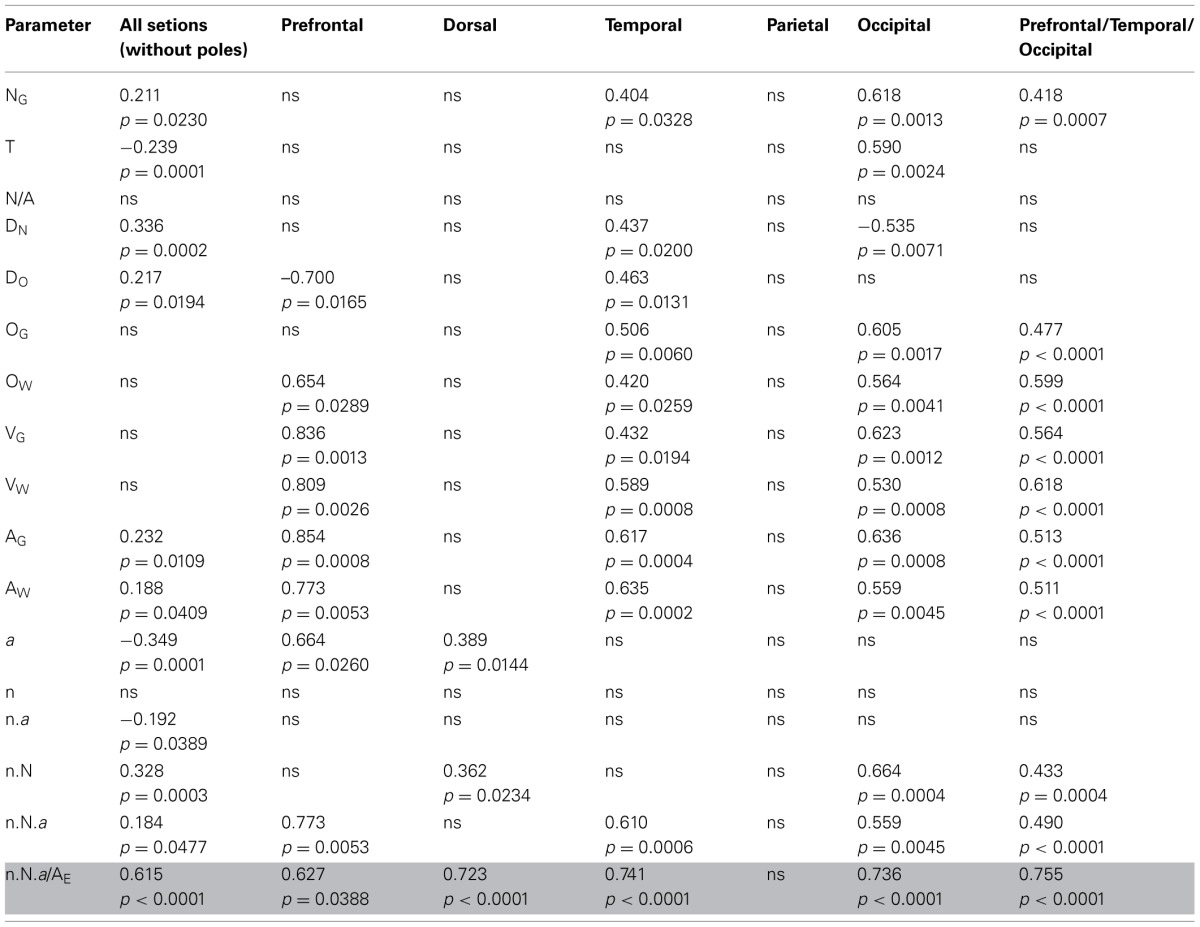
**Correlation between local GI and other parameters along the AP axis**.

Spearman correlation coefficients for GI and each of the parameters indicated. Correlations exclude the prefrontal, temporal, and occipital poles. ns, not significant at the p < 0.05 level. a, average fiber caliber in the white matter; n, estimated fraction of gray matter neurons connected through the white matter; n.a, product that, multiplied by N_G_, defines A_W_ according to our model (Mota and Herculano-Houzel, 2012); n.N, estimate proportional to the total number of fibers in the white matter that are connected to the gray matter; n.N.a, estimate of the surface area of the gray/white matter interface, A_W_; n.N.a/A_E_, estimate of the degree of folding of the gray/white matter interface.

## Discussion

The present quantitative analysis of the cellular composition, volume, surface area, thickness and gyrification of the human cerebral cortex across its anteroposterior axis reveals that 42% of the variation in the parameters analysed is explained jointly by variations in numbers of neurons, other cells, volume and surface area of both the gray and white matter. Principal component analysis shows that local variations are next influenced by cellular densities and position along the AP axis. Cortical folding, in turn, loads mostly the third component, while cortical thickness loads the fourth. Multivariate analysis thus supports our hypothesis that cortical folding and thickness are consequences of the distribution of neurons, and not determinants of it (Mota and Herculano-Houzel, 2012).

Here we show that, along the AP axis of the human cerebral cortex, neurons are neither distributed in a single relationship to local cortical gray matter volume, nor in multiple site-specific relationships. Rather, we find a clear division of the cerebral cortex in two zones of different neuron × volume relationships: one corresponds to the occipital cortex (although we cannot at this time infer a direct correspondence to the occipital lobe), and the other encompasses all non-occipital areas, including prefrontal cortex. Thus, contrary to the former notion that the human prefrontal cortex is unusual in comparison to other cortical areas, we find that it is the occipital cortex, not the prefrontal cortex, that is distinct from all other regions in how neurons build its gray matter. Both prefrontal and occipital cortex, in turn, differ from the remaining intermediate cortical areas in their neuronal relationship with the white matter, with less white matter per neuron in the occipital cortex than in prefrontal cortex, and more in the other areas than in these two; an estimated average axon caliber that is larger in occipital and smaller in prefrontal cortices than in the cortical areas in between; and a connectivity fraction that decreases along the AP axis. In contrast to the two different neuronal building rules that apply within the gray matter and three within the white matter of the human cerebral cortex, we find a single building rule relating other cell densities and the O/N ratio to neuronal densities across the cortical sheet.

One particular concern in interpreting the results of a study that is based on a single subject, particularly a 65-year-old individual, is interindividual variability, including variability in the possible effects of aging. Restricting this study to a single individual does not address the confounding variable of individual differences in the distribution of neurons along the cerebral cortex. However, we predict that the relationships uncovered here should remain regardless of variations in total numbers of neurons, cortical surface or volume and other parameters across individuals. Another possible confounding variable is that any age-related changes in neuronal composition possibly restricted to parts of the cortex, such as the prefrontal cortex, could in principle create artificial “zones” in the distribution of neurons along the cortex. Although studies restricted to a few cortical sites initially suggested that age-related thinning might differ markedly across cortical areas [reviewed in Raz and Rodrigue (2006)], a systematic study of the whole cortex recently showed that age-related thinning occurs almost across the entire cortical surface (Fjell et al., 2009). Although some sites within the prefrontal cortex did show more pronounced thinning, the overall age-related reduction in cortical thickness was found to be relatively minor, of about 10% between ages 20 and 60 years. It is unlikely, therefore, that the zones identified here result from effects of aging that are specific to the entirety of the prefrontal and occipital cortices, although such effects, as any other type of variation from other sources, cannot at the present time be ruled out. A much larger study of individual differences in the distribution of neurons across the cerebral cortex is now being planned.

### A single, common rule for non-neuronal cells

There is now evidence from almost 30 species that the rules that underlie the addition of non-neuronal (mostly glial) cells to the brain and its various structures have not varied much over at least 95 millions years (Herculano-Houzel, 2012a). This evidence is the common relationships between O/N and neuronal density and between numbers of other (glial) cells and structure mass shared across brain structures and mammalian species of various orders (Herculano-Houzel, 2011b). Here we show that all regions of the human cerebral cortex share the same relationship between neuronal and non-neuronal density as well as a common relationship between O/N and neuronal density. These findings support our hypothesis that glial cells are added to brain tissue by a common mechanism that is shared across structures and species whereby the average neuronal size is the main factor that determines the distribution of glial cells and thus the glia/neuron ratio in brain tissue (Herculano-Houzel et al., 2006; Herculano-Houzel, 2011b). As glial cells invade the cerebral tissue postnatally, higher glia/neuron ratios are established in regions previously occupied by larger average neuron sizes, such as the prefrontal cortex, and lower glia/neuron ratio are established where the average neuronal sizes are smaller, such as in the occipital cortex.

### Not a single gradient: implications for development

The AP gradients of neuronal density and number of neurons per unit surface area found here within the human cerebral cortex appear similar to those that have been described in the literature within non-human primate cortices (Collins et al., 2010; Cahalane et al., 2012). However, our systematic analysis of their relationship to other parameters such as local gray matter volume and thickness and white matter composition allowed a novel discovery that the previous studies could not have made. While those studies posited that neuronal density and surface density are organized as single gradients across the cortical surface, and indeed they might appear that way, we show that the human cerebral cortex is not structured as one uniform gradient in the anteroposterior axis. Rather, there are two gradients in what concerns neuronal density per volume of gray matter (one steep and one shallow gradient, related to two region-specific building rules for gray matter, one in occipital cortex and one in non-occipital cortex), and three gradients in what concerns neuronal surface density (related to three region-specific building rules relating gray and white matter, one in occipital cortex, one in prefrontal cortex, and one in the non-occipital, non-prefrontal areas). As a consequence of these gradients, average neuronal size (inferred from the inverse of neuronal density) is estimated to be largest in the prefrontal cortex, and smallest in the occipital cortex, varying along two consecutive gradients that are however continuous along the AP axis. This predicted variation in average neuronal cell size is supported by a documented decrease of dendritic arbor size along the anteroposterior axis of primate cortices, with the largest neurons in prefrontal cortex and the smallest in the occipital cortex (Elston et al., 2001; Jacobs et al., 2001).

A single gradient in neuronal volume and surface densities has been interpreted as evidence of a single isocortex-wide developmental pattern (Cahalane et al., 2012). Our finding that the apparently single gradient in fact breaks down into two very clear zones indicates that there is not a single isocortex-wide developmental pattern, but two. We speculate that these zones correspond to those zones specified by different morphogens in the developing cerebral cortex, expressed in gradients that might be related to those found here (Bishop et al., 2000; Fukuchi-Shimogori and Grove, 2001; O'Leary et al., 2007). Emx2 and Pax6, for instance, are known to specify the posterior portion of the cerebral cortex (Bishop et al., 2000), and other transcription factors such as COUP-TF1, Etv5, Etv4, Etv1, Sp8 and Met/Peg1 are also known to be expressed in AP gradients and to interfere in cortical specification (Sansom and Livesey, 2009). The morphogen FGF8 is another candidate molecule to participate in the division of the cerebral cortex into two “occipital and non-occipital” zones, given the rostral location of its source (Fukuchi-Shimogori and Grove, 2001) and its suppressing role on presumptive visual areas (Fukuchi-Shimogori and Grove, 2001; Garel et al., 2003; O'Leary et al., 2007; Sansom and Livesey, 2009).

The two-zone scenario that we propose does not rule out the model put forward by Barbara Finlay who posits that gradients in local neuronal densities are related to gradients in cortical neurogenesis (Finlay et al., 1998). However, while delayed neurogenesis would explain the generation of larger numbers of neurons in a developing cortical region, it does not necessitate that these neurons will be smaller and accumulated in larger densities. We stress that the distinct relationship between local gray matter volumes and numbers of neurons in the occipital cortex calls for the recognition that this is a distinct cortical zone, which is thus likely formed as the result of a distinct developmental program. Indeed, it has been suggested that elevated levels of neurogenesis are specific to the visual cortex in primates, which occupies the occipitalmost regions of the cortex (Dehay and Kennedy, 2007). It is thus necessary to search for additional mechanisms to account for (1) the distinct building rules between occipital and non-occipital cortical zones and (2) the formation of gradients in neuronal volume and surface densities within each zone. Gradients of morphogens are likely candidates to account for both (1) and (2), creating zones with distinct neurogenetic characteristics while sharing an internal patterning in gradients of neuronal volume and surface densities—although it is still unclear how these density gradients would be generated, or would be related to gradients in neurogenesis, unless the mechanisms that delay neurogenesis and thus lead to larger numbers of progenitor would also render these progenitors, as well as their descendant cells, necessarily smaller in size.

Although we could not address individual functional areas, the alignment of the local gray matter volume × numbers of neurons datapoints across all non-occipital cortical regions indicates that this relationship is shared across functional areas in this non-occipital zone. Indeed, our companion study of the distribution of neurons across functional areas of the mouse cerebral cortex shows that also in that species, there is a single relationship between the volume of a functional area and its number of neurons—except for the visual areas (Herculano-Houzel, Watson and Paxinos, see companion paper). Such shared relationships within each zone (occipital and non-occipital) have the remarkable implication that functional areas within each of these zones are not specified intrinsically by their neurons, but rather by their pattern of afferent and efferent connectivity.

We envisage that newborn neurons are first distributed equally within the cortical parenchyma of each zone (with local variations in density within each possibly according to a gradient in the timing of neurogenesis), effectively creating the parenchyma, which becomes segregated into functional areas as they form their connectivity. Our findings thus support a combination of the “protomap” and “protocortex” models of specification of cortical areas (Dehay and Kennedy, 2007), one in which two zones are specified in the gray matter that differ in how neurons are added to the developing cortex, followed by functional regionalization possibly defined both by patterns of intrinsic molecules and incoming thalamic afferents (O'Leary et al., 2007).

### White matter regionalization

While the gray matter is divided in two zones of different neuronal building rules, we find that connectivity patterns through the white matter split the human cerebral cortex into three recognizable zones: prefrontal cortex, occipital cortex, and all regions in between. It is noteworthy that two of these zones correspond to clusters of genetic influence on the interindividual variability of the area of the cortical surface (occipital cluster 12 and prefrontal clusters 2 and 4 in Chen et al. (2012). At this point we cannot determine whether these different zones are defined in response to afferent connectivity, such as thalamic inputs, or as a consequence of factors intrinsic to the gray matter, or both. Still, our data indicate that the human prefrontal cortex is regionally different from those areas posterior to it in its nearly two-fold larger fraction of gray matter neurons connected through the white matter (either sending or receiving axons through it), which is consistent with its predominantly associative role. Similarly, our results indicate that the occipital region of the cerebral cortex differs from those areas anterior to it in its two-fold smaller connectivity fraction, which is consistent with predominantly local processing of visual sensory information compared to other areas with more associative sensory roles. At the same time, the data indicate that the decrease in connectivity fraction along the AP axis within both prefrontal and occipital cortices is simultaneous to an increase in the average caliber of the fibers within the associated white matter, which is consistent with findings in the human and non-human primate corpus callosum (Aboitiz et al., 1992; Caminiti et al., 2009; Tomasi et al., 2012) and might partially compensate the longer propagation times due to the increased distances from posterior sensory areas to the callosum, although leaving prefrontal areas with longer expected propagation times. Because these areas are mostly associative in function, however, fast processing of inputs may not be as crucial as it is to the integration of sensory information.

Importantly, the recognition of three cortical zones that differ in their relationship with the white matter suggests that the factors that contribute to the specification of cortical zones must also lead to the regionalization of connectivity, affecting both the fraction of gray matter neurons that are connected through the white matter and the average caliber of these fibers. Identifying these factors and how they affect gray matter connectivity through the white matter could be a novel field of research in developmental neurobiology with important new outcomes for understanding developmental diseases.

### The non-uniformity of the cerebral cortex

Here we show that in the human cerebral cortex, as in other non-human primate cortices, the distribution of neurons underneath the surface is not constant as originally posited (Rockel et al., 1980), but rather varies by 3–4 fold. More importantly, our systematic analysis allows us to offer, for the first time, an explanation for this variation: that the N/A ratio varies locally depending on the connectivity fraction (n) and the average caliber of the fibers (*a*) at the gray/white matter surface, in agreement with our connectivity model of cortical folding (Mota and Herculano-Houzel, 2012). As the product n.*a* increases along the AP axis, so does the N/A, in what appears to be a single gradient, but turns out to be three, as discussed above. As observed by others (Rockel et al., 1980; Collins et al., 2010; Cahalane et al., 2012), N/A is maximal in the occipital cortex, which is predicted in our model as the area where both neuronal density and the product n.*a* are greatest. Additionally, the variation in N/A according to the local connectivity through the white matter (both n and *a*) in three different zones explains how neurons that are distributed in a common fashion across the cortical volume (for instance, within non-occipital cortex) become spread unevenly under the surface (between prefrontal and non-prefrontal, non-occipital cortex). As discussed in more detail in the companion study (Herculano-Houzel et al., see companion study), we propose that the volume of neurons distributed commonly across non-occipital areas becomes spread laterally in different manners across the three zones identified here depending on the local combinations of N, n, and *a*, thus leading to an apparently single gradient in N/A along the cortical surface.

A constant N/A has been a staple of models of cortical development, scaling and evolution (for instance, Rakic, 1988; Prothero, 1997; Zhang and Sejnowski, 2000; Karbowski, 2003). The recognition that N/A varies systematically along the cortical surface together with cortical connectivity through the white matter has important consequences for updating these models and thus reexamining the developmental mechanisms that underlie cortical evolution, in particular those leading to the formation of the human brain.

### Local variations in cortical folding

Although there is no consensus in the literature on what are the mechanisms leading to cortical folding (Hilgetag and Barbas, 2006), many models adopt explicitly or implicitly the notion that the cerebral cortex becomes more folded as a consequence of a lateral expansion of the cerebral cortex [reviewed in Welker (1990) and Kaas (2009)], which has been proposed to result in increasing number of neurons distributed in uniform cortical columns (Rockel et al., 1980; Rakic, 1988). By this logic, the degree of cortical folding across species, but also within a single cortex, is expected to vary as a simple function of numbers of neurons. Another factor that has been suggested to influence the degree of gyrification is mechanical resistance to folding of the gray matter related to its thickness, which might account for differences in the degree of folding across orders (Pillay and Manger, 2007), even though this opposes the positive correlation between gyrification and cortical thickness across species of larger brain size (Hofman, 1985).

In contrast to all these models, here we show that local variations in cortical folding are not explained by variations in cortical thickness. We note that the cortical thickness analyzed here is the average value for entire coronal sections, thus spanning different cortical areas with different thicknesses, which explains why the average values reported here disagree with thickness values observed in particular brain areas in other studies, such as the thin cortex around the central sulcus (Fjell et al., 2009). Still, any universal correlations that applied to cortical thickness, such as with gyrification, should still apply to the averages of coronal sections—but we find no such correlation. We also find that local variations in cortical folding are not a simple uniform function of local numbers of neurons. Rather, we find that local variations in cortical folding are correlated with variations in white matter volume, and are best described by variations in the estimated local folding of the white matter predicted by our connectivity-based model of cortical folding (Mota and Herculano-Houzel, 2012). Remarkably, this model, developed from the analysis of variations in cortical gyrification across mammalian species, thus seems to also apply within an individual cerebral cortex. Most importantly, our model explains variations in cortical folding throughout the human cerebral cortex as a continuous function of connectivity through the white matter, despite the finding that the pattern of connectivity (connectivity fraction and average fiber caliber) varies discontinuously across three different zones, as discussed above.

The consensus between our model and our experimental findings within the human cerebral cortex not only supports our model but also indicates strongly that the mechanisms that lead to the folding of an individual cortex in its development are the same that lead to greater or smaller degrees of gyrification across species, and thus possibly also across individuals of a same species. Our connectivity-based model of cortical folding is thus a good candidate for fundamenting future studies of the generation of cortical macroscopic morphology in brain development and evolution.

### Why are occipital and prefrontal cortices different?

Studies of the human cerebral cortex often point to parameters that appear different in the prefrontal cortex as candidates to explain our cognitive uniqueness, such as the spacing of pyramidal neurons within prefrontal area 10 (Semendeferi et al., 2011; Teffer and Semendeferi, 2012) and dendritic lengths in this area (Jacobs et al., 2001). Our systematic analysis of the neuronal distribution across the human cerebral cortex, however, suggests that these are simply expected findings along a common distribution that applies to non-occipital areas. Indeed, the anteriormost sections in our sample, in which we find the lowest neuronal densities, probably correspond to area 10 (Semendeferi et al., 2011)—but our analysis suggests that this characteristic is to be expected given the location of this area along the AP axis.

“Occipital” cortex in our analysis includes area V1, but is not limited to it, and the relatively small volume of this area [estimated at only 1.5% of the entire human gray matter (Frahm et al., 1984; Rilling and Insel, 1999; De Sousa et al., 2010), while “occipital” cortex here comprises 22.9% of all gray matter] suggests that the different relationships observed here for “occipital” cortex are not due to the inclusion of V1 but indeed represent a more widespread zone of visual areas that includes V1. Visual areas in the occipital cortex have long been known to differ from other cortical regions in their neuronal density (Rockel et al., 1980), and recent studies of gene expression confirm that V1 has a distinct gene expression profile compared to a limited number of other cortical areas in the macaque and human cortices (Bernard et al., 2012). In turn, our data suggest that the entire “occipital” cortex, extending in our sample for over 5 cm of the posteriormost cortex and including non-V1 visual areas, is organized in an entirely different fashion than the rest of the cortex, with far more neurons per volume than any other region. This interpretation is supported by the observation that visual areas other than V1 also have high neuronal densities (Collins et al., 2010).

Remarkably, we recently found that in non-human primates, area V1 alone, which corresponds to an average 12% of cortical gray mass, holds on average 36% of all cortical neurons (Collins et al., 2013)—a percentage of neurons that is very similar to that found here for the entire “occipital” cortex, which includes other visual areas. We have previously speculated that, for functional reasons related directly to increasing cortical size and the impossibility of extending indefinitely the size of functional areas, V1 fails to increase in absolute size above a certain limit in much larger brains (Collins et al., 2013), thus decreasing in *relative* size in the human cortex compared to great apes and other primates (Kaas, 2000), as its function becomes parcelated across a growing number of visual areas in larger cortices. The present finding that the human occipital cortex contains one third of all cortical neurons provides circumstancial evidence that the relative number of neurons dedicated primarily to visual processing remains similar across human and non-human primates, at around 33% of all cortical neurons, whether, for reasons related to the scaling of connectivity, those neurons are concentrated in one single visual area, a few, or several.

This division of the cortical gray matter into two zones of different neuronal distribution patterns is unlikely to be a distinctive characteristic of the human cortex; as shown in the companion study, a similar division of the gray matter in two zones, visual and non-visual, is also found in the mouse cortex (Herculano-Houzel, Watson and Paxinos, see companion paper), where the visual (occipital) areas are also outliers in the distribution of neurons across the cortical volume, and have the largest neuronal densities, just as in the human cortex. This suggests that occipital (visual) cortex is indeed a different cortical zone both in primates and in rodents, even though the molecular signatures identified in primate V1 were not observed in mouse visual cortex (Bernard et al., 2012). It is tempting to speculate that the reason why visual cortex is built with larger neuronal densities and thus smaller neurons is related to the particular properties of visual physiology, with very small dendritic fields allowing for the processing of information with high spatial and temporal resolution (Elston et al., 2001), although an earlier study failed to find a significant relationship between the tangential width of dendritic arbors and the width of the receptive field of V1 neurons in the cat (Martin and Whitteridge, 1984). Moreover, large neuronal densities do not seem to be an absolute requirement for cortical vision, as area V1 in the opossum has recently been found to have a neuronal density of only about 20,000 neurons/mg, which is smaller than in other sensory cortices of this marsupial, a highly visual species (Seelke et al., 2012).

If being distinctive for having “more neurons than expected” is akin to having “more volume than expected,” as has been argued consecutively for the human prefrontal cortical gray matter and then for the white matter, then one should, by the same logic, envision the possibility that the “most remarkable feature” of the human brain is its occipital cortex, and not its prefrontal counterpart. While the merit of this somewhat absurd possibility deserves further thought, it seems far more productive to consider that these are simply different zones of the cerebral cortex that are built differently due to developmental constraints and/or evolutionary paths.

Finally, we would like to draw attention to the small relative number of neurons located in the human prefrontal cortex (defined as all cortex situated anterior to the corpus callosum; Schoenemann et al., 2005): only 7.6% of all cortical neurons, which is even less than its relative mass of 10.0% of all cortical gray matter. Determining whether the relative number of cortical neurons in the prefrontal cortex is higher in human than non-human primates requires a comparison with other primate species that is in progress. However, the companion paper shows that the proportion of neurons in frontal areas available for purely associative functions in the mouse cortex is also 8%, as we found in the human prefrontal cortex (Herculano-Houzel, Watson and Paxinos, see companion paper). Together with the finding for occipital/visual cortex, this striking coincidence raises the possibility that the relative distribution of cortical neurons across areas of similar general functions—visual, associative, and possibly others—is similar across mammals as different as rodents and primates. In that case, human evolution—as that of other species—would not have entailed a relative expansion of the prefrontal cortex (at least not in what regards relative numbers of neurons), contrary to what is still a predominant view in the field.

So far, we consider that the most likely explanation for the remarkable cognitive abilities of the human brain lies simply in its very large, absolute number of neurons, both in the brain and cerebral cortex as a whole (Herculano-Houzel, 2009, 2012a). Even if at a putatively constant percentage of all cortical neurons, this would amount to the largest number of prefrontal, associative neurons in the brain of any species (Herculano-Houzel, 2009, 2012a). Determining whether the human prefrontal cortex (or the occipital cortex, for that matter) is extraordinary in its cellular composition and relative number of neurons will require comparing it to the distribution of cells and tissue in the prefrontal (or occipital) cortex of other primates. While this task would be currently unfeasible if it depended on anatomical criteria, the systematic analysis of the distribution of cells and tissue along the AP axis undertaken here makes it possible. That study is now underway.

### Conflict of interest statement

The authors declare that the research was conducted in the absence of any commercial or financial relationships that could be construed as a potential conflict of interest.
