# Supplementary material for: The human cerebral cortex is neither one nor many: neuronal distribution reveals two quantitatively different zones in the gray matter, three in the white matter, and explains local variations in cortical folding
Source: Front Neuroanat. 2013 Sep 2;7:28. doi: 10.3389/fnana.2013.00028 (PMC3759024; doi:10.3389/fnana.2013.00028)
Supplement: Supplementary file 1 [file DataSheet1.DOCX]

**Supplementary information**

Calculation of AG, AW, VG and VW from coronal measurements along the AP axis

One can obtain approximate formulas for the total grey and white matter surface areas A_G_ and A_W_ and volumes V_G_ and V_W_ for each section in a series of coronal areas and perimeters S_0_, S_1_, S_2_... and P_0_, P_1_, P_2_… To see how this can be done, first consider that, in general, each contour can be approximated with arbitrary precision by some polygon. Let vectors **r**_n_^i^ be the *i*-th vertex of such a polygonal contour in section *n*; and let **d**_n_^i^= **r**_n_^i+1^ – **r**_n_^i^ be the *i*-th edge of the same contour. Likewise, **r**_n+1_^i^ and **d**_n+1_^i^ are the *i*-th vertex and edge of the contour in the adjacent section *n+1*. Then, P_n_ = Σ|**d**_n_^i^| and S_n_ = Σ S_n_^i^ = ^1^/_2_Σ|**d**_n_^i^×**r**_n_^i^|, where the sums are over all values of *i*.

The best estimate of the volume and lateral surface area of the section between contours *n* and *n+1* will in general depend non-trivially on the position of all vertexes, and can only be computed algorithmically. But it is possible to approximate both quantities by simple functions of perimeters P_n_ and P_n+1_ and coronal areas S_n_ and S_n+1_, provided that we assume that (a) each section’s lower contour is approximately congruent to that of its neighbors and that (b) the slice’s lateral surface makes an approximately constant angle with the coronal plane. In this case, it can be shown that

A_n_ = {(S_n+1_-S_n_)^2^ + [h(P_n_+P_n+1_)/2]^2^}^1/2^

V_n_ = h[S_n_+S_n+1_+(S_n_.S_n+1_)^1/2^]/3

where h = 2 mm is the section thickness. Total values of A_G_, A_W_, V_G_ and V_W_ are obtained by summing over all values of *n*.

To see how this is done, consider the total section lateral area A_n_=Σ A_n_^i^ and volume V_n_=Σ V_n_^i^, where A_n_^i^ and V_n_^i^ are respectively the element of lateral area and volume of the polyhedron defined by points **r**_n_^i+1^, **r**_n_^i^ **r**_n+1_^i+1^ and **r**_n+1_^i^; plus the freely chosen origin of coordinates for each contour, **c**_n_ and **c**_n+1_ (let also **h**=**c**_n_ – **c**_n+1_ such that |**h**|=h). We can then write L_n_ as the sum over all i of the sum of the areas of two triangles defined by {**r**_n_^i^_,_ **r**_n+1_^i+1^, **r**_n+1_^i^} and {**r**_n_^i^_,_ **r**_n_^i+1^, **r**_n+1_^i^}. But hypothesis (a) implies that or each n both triangles are co-planar, so that, for the lateral area,

A_n_=^1^/_2_Σ |**d**_n+1_^i^×(**h** + **r**_n+1_^i^ – **r**_n_^i^)+ **d**_n_^i^×(**h** + **r**_n+1_^i+1^ – **r**_n_^i+1^)|

Recalling that **h** is perpendicular to **v**_n_^i^, this can be rewritten as

A_n_=^1^/_2_Σ | (**d**_n+1_^i^×**r**_n+1_^i^ – **d**_n_^i^×**r**_n_^i^)**û_z_** + h(|**d**_n_^i^|+|**d**_n+1_^i^|)**û_r_**|,

where **û_z_** and **û_r_** are respectively the vertical and radial unit vectors. But hypothesis (b) implies that the ratio between the vertical and radial components in the terms of the expression above is the same for all *n*, and thus we can perform the summation before taking the modulus. Recalling the expressions for S_n_ and P_n_, we then obtain

A_n_= | (^1^/_2_Σ**d**_n+1_^i^×**r**_n+1_^i^ - ^1^/_2_Σ **d**_n_^i^×**r**_n_^i^)û_z_ + h/2(Σ|**d**_n_^i^|+Σ|**d**_n+1_^i^|) û_r_|

A_n_= | (S_n+1_ - S_n_)û_z_ + h/2(P_n_ + P_n+1_) û_r_|

A_n_ = {(S_n+1_-S_n1_)^2^ + [h(P_n_+P_n+1_)/2]^2^}^1/2^

To obtain the expression for the *n*-th sectional volume, we use hypothesis (a) to write it as the volume of a right truncated pyramid with the upper and lower bases given by the two adjacent congruent contours *n* and *n+1*. This is in turn the difference between the volumes of two congruent pyramids, with respectively contours *n* and *n+1* as bases, and heights H and H-h. Thus,

V_n_ =[S_n_H - S_n+1_(H - h)]/3

Furthermore, congruence requires that H/(H-h) = (S_n+1_/S_n_)^1/2^, and therefore
H = h[S^1/2^_n+1_/( S^1/2^_n+1_-S^1/2^_n_)]. Substituting H in the expression for V_n_, we finally get

V_n_ =h[(S_n_ - S_n+1_) [S^1/2^_n+1_/( S^1/2^_n+1_-S^1/2^_n_) + S_n+1_]/3

V_n_ = h[S_n_+S_n+1_+(S_n_.S_n+1_)^1/2^]/3
